# Integrated Analysis of Prognostic and Immune Associated Integrin Family in Ovarian Cancer

**DOI:** 10.3389/fgene.2020.00705

**Published:** 2020-07-17

**Authors:** Anqi Wu, Sai Zhang, Jiaqi Liu, Yifeng Huang, Wenyu Deng, Guang Shu, Gang Yin

**Affiliations:** ^1^Department of Pathology, Xiangya Hospital, Central South University, Changsha, China; ^2^Deparment of Pathology, School of Basic Medicine Sciences, Central South University, Changsha, China; ^3^Department of Anesthesia, School of Medicine, Central South University, Changsha, China; ^4^Departmemt of Nursing, School of Nursing, Central South University, Changsha, China

**Keywords:** integrin, ovarian cancer, prognosis, TIL, metastasis, drug-resistance

## Abstract

Human integrin receptors are important for cell-cell and cell-matrix adhesion in normal epithelial cells. Emerging evidences have indicated integrin members are involved in cancer development and progression as well. However, the expression patterns and clinical significance of the whole integrin family in ovarian cancer (OC) have not yet been well understood. In the present study, we utilized the public datasets including GEPIA, GEO, ONCOMINE, cBioPortal, Kaplan-Meier Plotter, TIMER databases, to analyze the expression and prognostic value of integrin members in OC. We found ITGA3/B4/B6/B7/B8 were abnormally overexpressed in OC; ITGA6 was good prognosis predictor in OC; ITGA3/ B4/B8 were poor prognosis predictor specially in advanced OC patients; elevated ITGA3/B4 might promote metastasis and elevated ITGA3/B8 might promote platinum resistance of OC; ITGA3 and ITGB4 might synergistically or independently regulate cell adhesion and proliferation; ITGA4/AL/AM/AX/B2/B7 showed strong correlations with various tumor immune infiltrates (TILs), especially with pro-tumor immunes cell types like monocyte, M2 macrophage and exhaustion T cells infiltration; ITGAL/AM/B2/B7 and residing memory CD8+ T cells marker ITGAE were specially associated with early OC patients outcome. Our results implied that ITGA3/B4 were important prognostic markers of advanced OC, ITGAL/AM/ B2/B7 were immune associated prognosis markers of early OC, together they might render important therapeutic targets for OC.

## Introduction

Ovarian cancer (OC) as the most lethal gynecological cancer, its mortality rate ranks fifth in female's death associated with cancer (Siegel et al., 2019). Patients diagnosed with OC are at a median age of around 60 years, and majority of them are diagnosed at advanced stages, with companion of metastasis. Lacking of effective early stage detection greatly contributes the poor survival rate of OC. Surgical debulking and platinum-based chemotherapy are the first-line therapy strategy for OC treatment. Although most patients show good responses to chemotherapy for the first time, recurrence probably develop within 18 months, and eventually result in succumbing to cancer (Jayson et al., 2014; Torre et al., 2018; Lheureux et al., 2019). Thus, effective indicators for early diagnostic, strategies for solving metastasis and chemotherapy resistance, or other novel treatments like immunotherapy are urgently needed to improve the prognosis of OC patients (Bast, 2003).

As so far, integrin family contains 18 alpha subunit genes (ITGA1, ITGA2, ITGA3, ITGA4, ITGA5, ITGA6, ITGA7, ITGA8, ITGA9, ITGA10, ITGA11, ITGA2B, ITGAD, ITGAE, ITGAL, ITGAM, ITGAV, ITGAX) and 8 beta subunit genes (ITGB1, ITGB2, ITGB3, ITGB4, ITGB5, ITGB6, ITGB7, ITGB8). The heterodimers formed by α and β subunit non-covalent binding, function as adhesion receptors. Integrins widely distribute on the cell surface of normal epithelial cells. By recognizing specific extracellular ligands, integrins change the conformation and activate certain intracellular signaling to mediate cell-cell adhesion, cell-matrix adhesion, respond to microenvironmental signals and regulate cell mobility and proliferation (Ruoslahti and Pierschbacher, 1987; Schwartz et al., 2018). Emerging evidence have shown integrins also play important roles in angiogenesis and cancer development, including cell migration, apoptosis, proliferation and stemness (Silva et al., 2008; Winograd-Katz et al., 2014; Seguin et al., 2015).

ITGA5, ITGAV, ITGB1, and ITGB3 are the most popular studied integrin members in OC, which can form α5β1, αvβ1, αvβ3 heterodimeric receptors. Most literature support they function as oncogenic genes as promoting adhesion, migration and proliferation in OC (Cruet-Hennequart et al., 2003; Sawada et al., 2008; Ruseva et al., 2009; Mitra et al., 2011; Ohyagi-Hara et al., 2013; Xue et al., 2013; Gong et al., 2016; Shinderman-Maman et al., 2016; Villegas-Pineda et al., 2017). However, some studies have reported higher expression of ITGB3 or ITGAV negatively associate with prognosis (Partheen et al., 2008, 2009) and invasion (Kim et al., 2008; Chen et al., 2009, 2016; Kaur et al., 2009), suggesting they may be tumor suppressors in OC. Meantime, studies on other integrin members like ITGB2/7 and most of ITGAs remain sparse. It's important to figure out which integrin members are crucial and how they function in OC. Thus, we analyzed the expression of each integrin member in OC, and their relationship with prognosis, genetic alteration, metastasis, drug resistance, and immune infiltration, combined with exploring of the possible mechanism of certain crucial integrins, we provide an insight into the potential biological functions and prognostic value of integrins in OC.

## Materials and Methods

### GEPIA2 (Gene Expression Profiling Analysis) Database

GEPIA2 is an interactive web server developed by Zhang's lab, helped biologists to explore the large TCGA and GTEx datasets. GEPIA2 provides multiple functions including gene expression comparison with tumor/ normal, various cancer type or pathological stage, correlation analysis between genes or signatures, survival analysis, isoform analysis, similar genes detection and dimensionality reduction (Tang et al., 2019). For the Expression in Box Plot, the threshold of |log2FC| was 1, *p*-value cutoff was 0.05, columns in red color represented tumor, columns in blue color represented normal. For the Correlation analysis, the correlation coefficient calculation method was Spearman correlation. Statistical significance with *p* < 0.05 was indicated with ^*^p < 0.01 was indicated with ^**^p < 0.001 were indicated with ^***^ and *p* < 0.0001 were indicated with ^****^.

### GEO (Gene Expression Omnibus) Datasets

GEO is a public functional genomic data repository contains numerous array and sequence-based study data. After searching the GEO database associated with ovarian cancer, GSE26712 (Bonome et al., 2008) was selected to validate GEPIA tumor/normal comparison results; GSE30587 (Brodsky et al., 2014) and GSE131978 GPL96 (Tassi et al., 2019) were selected to analyze the association of integrin members with ovarian cancer metastasis; GSE30161 (Ferriss et al., 2012), GSE51373 (Koti et al., 2013) and GSE131978 GPL570 (Tassi et al., 2019) were selected to analyze the association of integrin members with ovarian cancer chemoresistance. The data of selected GEO datasets were downloaded and reanalyzed with Graphpad Prism 8.0.1. Data from GSE30587 (Brodsky et al., 2014) which contained paired metastasis and primary samples were analyzed with paired *t*-test, others were analyzed with unpaired *t*-test, statistical significance with *p* < 0.05 was indicated with ^*^p < 0.01 was indicated with ^**^p < 0.001 were indicated with ^***^ and *p* < 0.0001 were indicated with ^****^.

### Oncomine Database

Oncomine (http://www.oncomine.org) research edition contains 715 datasets and 86,733 samples, able to compute gene expression signatures, clusters and gene-set modules with robust analysis methods. We analyzed the transcriptional expression of integrin members between cancer tissues and their corresponding normal controls by Students' *t*-test. Cut-off of *p*-value and fold change were defined as 0.01 and 1.5 respectively. The setting of gene rank was all, data type was mRNA.

### cBioPortal

cBioPortal (http://www.cbioportal.org/) is an open platform for comprehensively exploring and visualizing multidimensional cancer genomics data (Cerami et al., 2012; Gao et al., 2013). In this study, the ovarian serous cystadenocarcinoma (TCGA, Firehose Legacy) dataset including data from 594 patients was selected for further genetic alteration analysis. Mutations, Putative copy-number alterations from GISTIC and mRNA Expression z-Scores (RNA Seq V2 RSEM) were selected for genomic profiles. The correlations between mRNA expression of each integrin member and their CNA (relative liner copy-number values) or protein (mass spectrometry by CPTAC) were plotted. The co-expression gene lists of ITGA3 and ITGB4 were downloaded for later study.

### Kaplan-Meier Plotter [Ovarian Cancer]

The online Kaplan-Meier plotter (http://www.kmplot.com) was used to assess the effect of each integrin family member on ovarian cancer prognostic (Gyorffy et al., 2012). The TCGA dataset (*n* = 565) was chosen for the OS analysis and PFS analysis, GSE30161 (Ferriss et al., 2012), GSE26193 (Mateescu et al., 2011; Gentric et al., 2019; Kieffer et al., 2020) and GSE14764 (Denkert et al., 2009) were chosen for external validation datasets. The ovarian cancer patients were split into high and low expression groups by mRNA expression of auto-selected best cutoff. The Kaplan-Meier survival plot showed Hazard ratio (HR), 95% confidence intervals (CI) log-rank *p*-value for each corresponding gene, and the number at risk below each plot. Cutoff of *p* < 0.05 was considered as statistical significance.

### TIMER (Tumor Immune Estimation Resource)

TIMER (https://cistrome.shinyapps.io/timer/) is an interactive web server that can systematically analyze immune infiltrates in cancer with its own algorithm (Li et al., 2016, 2017). We analyzed the correlation of each integrin member with the abundance of immune infiltrates in OC, including B cells, CD4+ T cells, CD8+ T cells, Neutrophils, Macrophages, and Dendritic cells, by gene modules. In addition, we also analyzed the association of each integrin member and gene markers of tumor-infiltrating immune cells by correlation modules. The gene markers of tumor-infiltrating immune cells listed in **Table 4** were reported in previous studies (Sousa and Määttä, 2016; Danaher et al., 2017; Siemers et al., 2017). The correlation analysis is the Spearman's correlation, statistical significance with *p* < 0.05 was indicated with ^*^p < 0.01 was indicated with ^**^p < 0.001 were indicated with ^***^ and *p* < 0.0001 were indicated with ^****^.

### Gene Ontology (GO) and Kyoto Encyclopedia of Genes and Genomes (KEGG) Enrichment

The Database for Annotation, Visualization and Integrated Discovery (DAVID) (https://david.ncifcrf.gov/) is a powerful tool to investigate functional annotation of a certain gene list (Huang da et al., 2009a,b). The genes showed correlation coefficient (r) >0.3 with ITGB4/8 were analyzed for GO and KEGG enrichment by DAVID. GO analysis included biological processes (BP), cellular components (CC), and molecular functions (MF). Top 10 significant enrichments of BP, CC, MF (*p* < 0.05 and rank by *p*-value) and all significant KEGG enrichments (*p* < 0.05) were visualized with ImageGP (http://www.ehbio.com/ImageGP/).

### UniHI

UniHI 7 (http://193.136.227.168/UniHI/pages/unihiSearch.jsf) integrates both protein-protein physical interaction and regulatory transcriptional interaction data from various data resources, such as HPRD, BIOGRID, INTACT, REACTOME, miRTarBase and TRANSFAC (Kalathur et al., 2014). In addition, UniHI 7 also integrates 4,203 different drugs and their corresponding targets (2139) information from DrugBank, so it can directly highlight the known drug target in the network contains PPI and regulatory interaction. The network of ITGA3 and ITGB4 was constructed by UniHI, and the interactions between integrin alpha subunit and beta subunit was also retrieved from UniHI.

## Results

### The Aberrant Expression of Integrin Genes in Ovarian Cancer Patients

**Figure 1:**
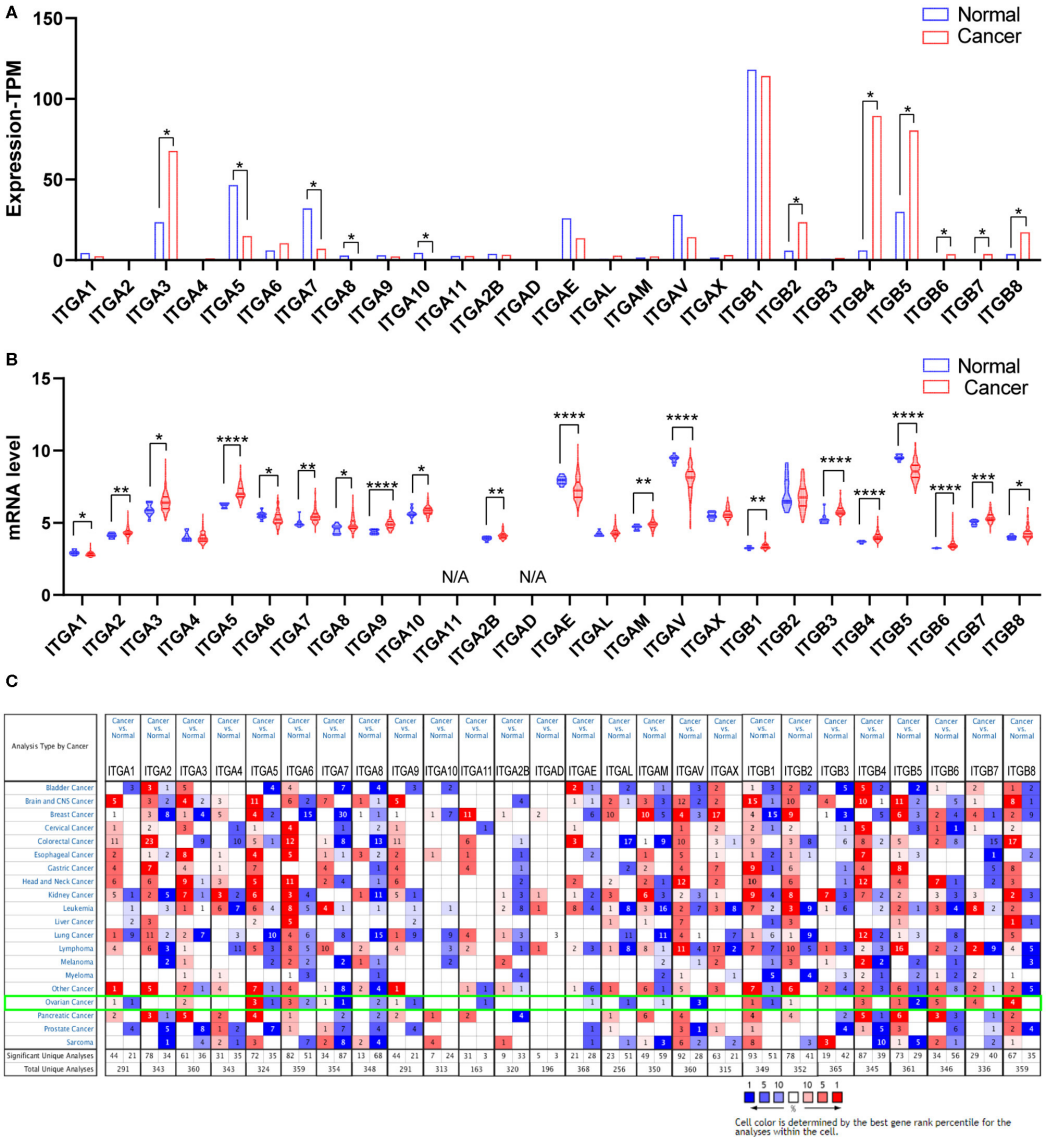
Expression of integrin members in normal ovary and ovarian cancer tissue. **(A)** The transcriptional expression from GEPIA database, *p < 0.05, number (cancer) = 426, number (normal) = 88. **(B)** The transcriptional expression from GEO database GSE26712, *p < 0.05, **p < 0.01, ***p < 0.001, ****p < 0.0001. number (cancer) = 185, number (normal) = 10. **(C)** The transcriptional expression from ONCOMINE database, threshold setting was *p*-value: 0.01, fold change: 1.5, gene rank: all, data type: mRNA.

Malignant tumor development requires various oncogene activation and suppressor inactivation. To understand the role of integrin genes played in ovarian cancer, we started with a comparison of integrin genes transcriptional expression between OC samples from TCGA (The Cancer Genome Atlas) and autopsy normal ovarian sample from GTEx (Genotype Tissue Expression) by GEPIA2 (Gene expression Profiling Analysis). The results indicated that ITGA3 and ITGB2/B4/B5/B6/B7/B8 transcriptional levels were significantly higher in OC samples than in normal ovary tissue, while ITGA5/A7/A8/A10 expression levels were significantly lower and other integrins showed no statistic differences (Figure 1A).

We further queried the expression of integrin genes in OC and normal tissue from GEO (Gene Expression Omnibus). Data analysis from GSE26712 (Bonome et al., 2008) suggested that ITGA2/A3/A5/A7/A8/A9/A10/A2B/AM and ITGB1/B3/B4/B6/B7/B8 mRNA expressions were significantly higher in OC tissue compared to HOSE (human ovarian surface epithelium). While ITGA1/A6/AE/AV and ITGB5 mRNA expression was significantly lower in OC tissue (Figure 1B).

In addition, we also explored the expression of integrin genes in various cancers compared to their normal tissues with the ONCOMINE database which compiled numerous independent studies with different patient cohorts (Figure 1C). One or more datasets showed significantly higher mRNA expressions of ITGA3 and ITGB2/B4/B6/B7/B8 in OC tissue, while ITGA8/A11/AE/AL/AM/AV showed significantly lower expression in OC tissue. However, some studies showed ITGA1/A5/A6/A7/A9 and ITGB1/B5 were statistically significantly higher expressed in OC but others showed the opposite. The specific results were shown in Table 1.

**Table 1:** Significant expression changes of integrin genes in transcriptional level between OC and normal tissue (Oncomine).

**Gene**	**Type of ovarian cancer**	**Fold change**	***p*-value**	***t*-Test**	**References**
ITGA1	Ovarian serous adenocarcinoma	−5.46	1.55E-10	−10.15	Yoshihara et al., 2009
	Ovarian serous surface papillary carcinoma	4.40	1.39E-04	4.18	Welsh et al., 2001
ITGA2	N/A	N/A	N/A	N/A	N/A
ITGA3	Ovarian serous adenocarcinoma	2.15	6.67E-06	5.20	Yoshihara et al., 2009
	Ovarian serous surface papillary carcinoma	2.00	2.00E-03	4.04	Welsh et al., 2001
ITGA4	N/A	N/A	N/A	N/A	N/A
ITGA5	Ovarian serous adenocarcinoma	−7.25	6.42E-10	−9.14	Yoshihara et al., 2009
	Ovarian clear cell adenocarcinoma	1.56	5.43E-05	6.62	Lu et al., 2004
	Ovarian carcinoma	1.80	1.35E-13	14.75	Bonome et al., 2008
	Ovarian serous surface papillary carcinoma	4.15	5.29E-04	3.67	Welsh et al., 2001
ITGA6	Ovarian serous adenocarcinoma	−3.87	5.62E-08	−8.15	Yoshihara et al., 2009
	Ovarian serous cystadenocarcinoma	−2.52	1.95E-04	−6.10	TCGA
	Ovarian mucinous adenocarcinoma	2.49	6.76E-04	4.25	Lu et al., 2004
	Ovarian serous adenocarcinoma	1.52	5.00E-03	3.35	Adib et al., 2004
	Ovarian mucinous adenocarcinoma	1.65	3.74E-05	9.45	Hendrix et al., 2006
ITGA7	Ovarian serous adenocarcinoma	−9.09	1.19E-20	−15.39	Yoshihara et al., 2009
	Ovarian carcinoma	1.51	6.29E-07	7.55	Bonome et al., 2008
ITGA8	Ovarian serous adenocarcinoma	−13.35	4.98E-07	−6.04	Yoshihara et al., 2009
	Ovarian serous cystadenocarcinoma	−1.92	2.00E-03	−4.09	TCGA
ITGA9	Ovarian serous adenocarcinoma	−3.30	5.26E-08	−7.02	Yoshihara et al., 2009
	Ovarian serous surface papillary carcinoma	4.40	1.39E-04	4.18	Welsh et al., 2001
ITGA10	N/A	N/A	N/A	N/A	N/A
ITGA11	Ovarian serous adenocarcinoma	−12.67	5.59E-12	−8.97	Yoshihara et al., 2009
ITGA2B	N/A	N/A	N/A	N/A	N/A
ITGAD	N/A	N/A	N/A	N/A	N/A
ITGAE	Ovarian carcinoma	−1.59	3.60E-06	−6.07	Bonome et al., 2008
ITGAL	Ovarian serous surface papillary carcinoma	−12.33	2.59E-06	−5.63	Welsh et al., 2001
ITGAM	Ovarian carcinoma	−4.99	1.00E-03	−4.28	Bonome et al., 2008
ITGAV	Ovarian carcinoma vs. normal	−2.86	7.18E-14	−13.51	Bonome et al., 2008
	Ovarian serous surface papillary carcinoma	−3.31	1.55E-05	−5.86	Welsh et al., 2001
	Ovarian serous adenocarcinoma	−1.97	4.00E-05	−4.75	Yoshihara et al., 2009
ITGAX	N/A	N/A	N/A	N/A	N/A
ITGB1	Ovarian serous cystadenocarcinoma	1.57	1.23E-06	11.10	TCGA
	Ovarian serous adenocarcinoma	−3.67	1.30E-09	−9.72	Yoshihara et al., 2009
ITGB2	Ovarian serous surface papillary carcinoma	6.89	6.31E-05	4.47	Welsh et al., 2001
ITGB3	N/A	N/A	N/A	N/A	N/A
ITGB4	Ovarian mucinous adenocarcinoma	1.59	1.27E-07	9.11	Hendrix et al., 2006
	Ovarian clear cell adenocarcinoma	1.56	3.00E-03	3.68	Hendrix et al., 2006
	Ovarian carcinoma	2.13	6.16E-10	9.84	Bonome et al., 2008
ITGB5	Ovarian serous cystadenocarcinoma	1.73	8.64E-07	10.10	TCGA
	Ovarian carcinoma	−2.02	2.40E-13	−13.30	Bonome et al., 2008
	Ovarian serous adenocarcinoma	−2.01	3.74E-05	−4.73	Yoshihara et al., 2009
ITGB6	Ovarian mucinous adenocarcinoma	2.56	9.77E-04	4.39	Lu et al., 2004
	Ovarian clear cell adenocarcinoma	2.14	3.00E-03	4.12	Lu et al., 2004
	Ovarian serous adenocarcinoma	1.97	1.80E-04	4.24	Lu et al., 2004
	Ovarian endometrioid adenocarcinoma	2.28	2.00E-03	3.86	Lu et al., 2004
	Ovarian serous surface papillary carcinoma	1.58	8.00E-03	2.57	Welsh et al., 2001
ITGB7	Ovarian serous adenocarcinoma	2.25	5.81E-06	5.16	Yoshihara et al., 2009
	Ovarian clear cell adenocarcinoma	1.54	2.00E-03	3.74	Hendrix et al., 2006
	Ovarian endometrioid adenocarcinoma	1.57	2.00E-03	6.06	Hendrix et al., 2006
	Ovarian serous adenocarcinoma	1.59	2.00E-03	6.43	Hendrix et al., 2006
ITGB8	Ovarian serous cystadenocarcinoma	2.42	6.17E-10	14.73	TCGA
	Ovarian serous adenocarcinoma	1.93	8.08E-06	6.86	Lu et al., 2004
	Ovarian endometrioid adenocarcinoma	1.50	5.00E-03	3.05	Lu et al., 2004
	Ovarian serous adenocarcinoma	8.15	2.77E-06	7.41	Yoshihara et al., 2009

Taken together, it was more reliable to conclude the ITGA3 and ITGB4/B6/B7/B8 abnormally overexpressed in OC patients.

### Genetic Alteration of Integrin Genes in Ovarian Cancer

**Figure 2:**
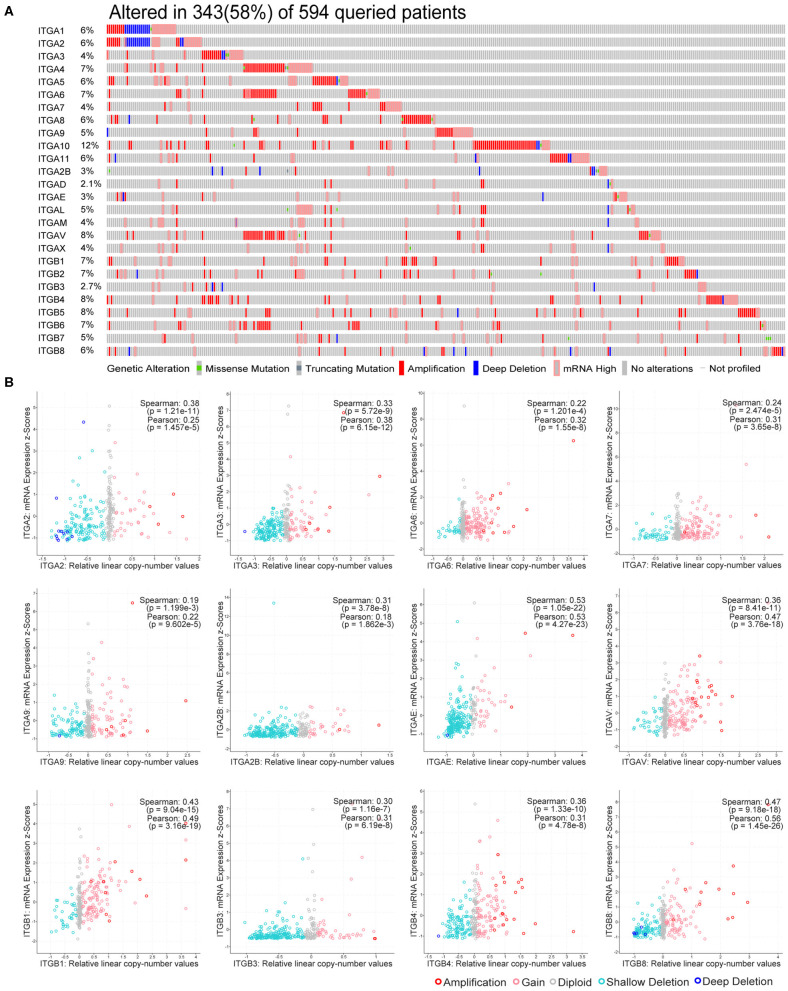
Genetic alteration of integrin genes in OC. **(A)** Genetic alteration percentage of integrin genes in OC. **(B)** Correlation of each integrin gene mRNA level and linearized copy-number alteration.

Genetic alteration and transcriptional regulation result in mRNA expression alteration. Genetic alteration of integrin genes in OC were analyzed with cBioportal (TCGA, Firehose Legacy). Integrin genes altered in 343 samples from 594 ovarian cancer patients (58%), of which ITGA10 genes accounted for the highest alteration frequency (12%; Figure 2A). We found that genetic missense mutation was rare, while amplification and mRNA alteration more frequently occurred in OC patients. Furthermore, we found ITGAE/AV and ITGB1/B8 mRNA expression were moderately correlated with their relative linear copy number alteration (CNA) value, ITGA2/A3/A6/A7/A9/A2B and ITGB3/B4 mRNA expression showed weak correlations with their relative linear CNA value (Figure 2B). Within the OC sample analyzed, most of integrin genes protein expression were in good correlation with their mRNA expression. ITGA2/A3/A6/AL and ITGB2/B4 mRNA level showed strong correlations with their protein level, ITGA1/A4/A5/A9/A11/AM/AV/AX and ITGB1/B3/B5/B6/B8 mRNA showed moderate correlations with their protein level, ITGA7 mRNA showed weak correlations with its protein level, ITGA2B showed no correlation between mRNA and protein level, protein level of ITGA8/A10/AD/AE and ITGB7 were not available (Supplementary Figure 1).

These results indicate integrin genes occasionally occurred with amplification and mRNA alteration in OC patients. The genetic alteration such as CNA partially led to the expression alteration of integrin genes, and the effect of post-transcriptional modification in most of integrins was minor.

### Prognostic Value of Integrin Genes in Ovarian Cancer

**Figure 3:**
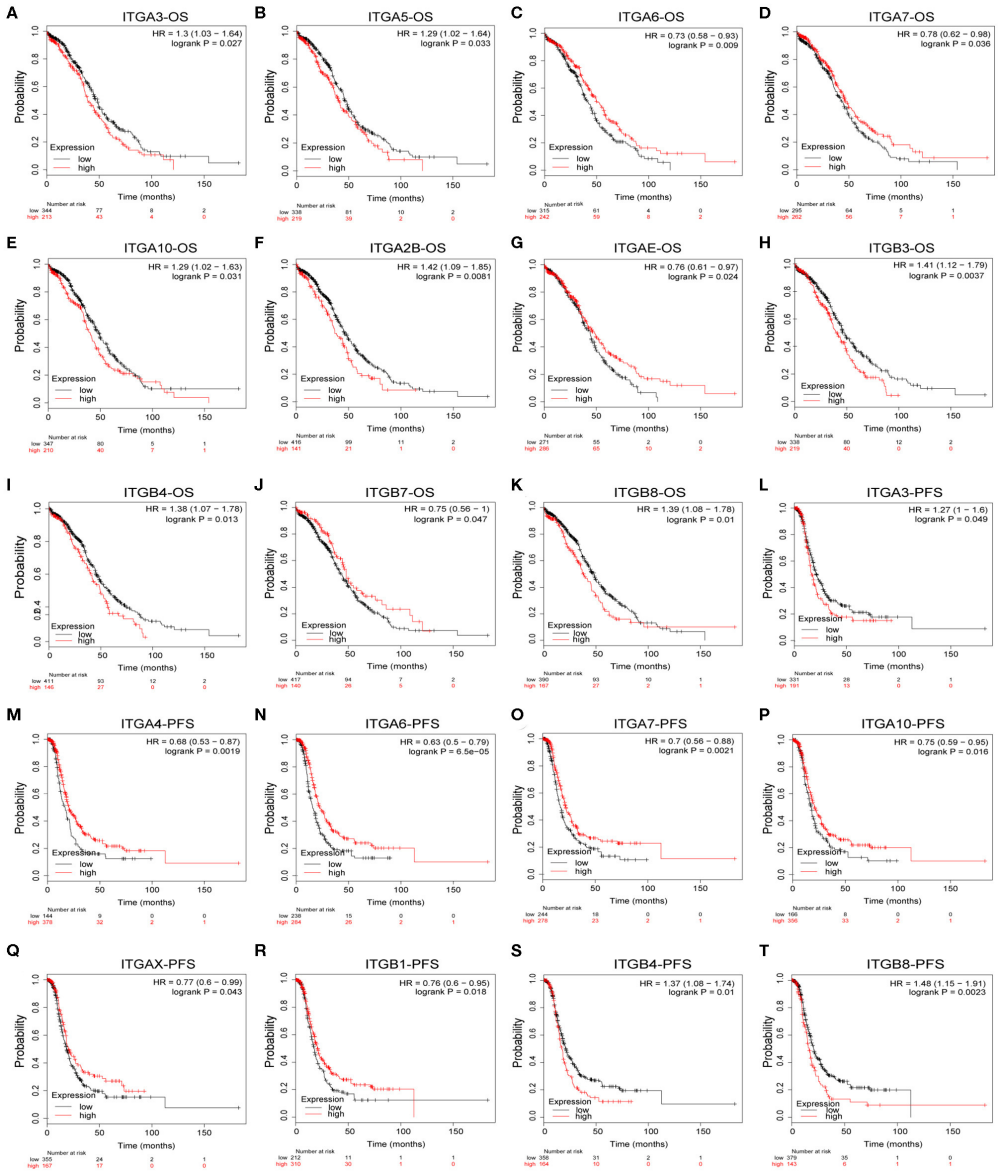
Prognostic value of integrin genes mRNA in OC. **(A–K)** Integrin genes showed significant correlations with OS in OC. **(L–T)** Integrin genes showed significant correlations with PFS in OC.

To explore the potential prognostic value of integrin genes in ovarian cancer, the Kaplan-Meier plotter tool was used to perform the survival analysis in 565 TCGA ovarian cancer samples with complete survival and integrin gene transcriptional expression data. Among all analyzed integrin genes, we found increased ITGA3/A5/A10/A2B and ITGB3/B4/B8 expressions were significantly associated with poor OS, while decreased ITGA6/A7/AE and ITGB7 expression were significantly associated with poor OS in ovarian cancer patients (Figures 3A–K). Meanwhile, higher mRNA expression of ITGA3 and ITGB4/8 were significantly associated with poor PFS, lower mRNA expression of ITGA4/A6/A7/A10/AX and ITGB1 were significantly associated with poor PFS in ovarian cancer patients (Figures 3L–T). To avoid the potential clinical outcome deviation caused by treatment, we further analyzed the prognostic value of integrin after excluding the minority patients without chemotherapy. After all, our results parallel showed ITGA5/B3/B4/B8 were significant risk factors for OS, ITGA6/AE were significant protective factors for OS; ITGA3/B4/B8 and additional ITGB6 were significant risk factors for PFS, ITGA4/A6/A7/A10/B1 were significant protective factors for PFS in OC patients received chemotherapy (Supplementary Table 1). Additionally, GSE26193 (Mateescu et al., 2011; Gentric et al., 2019; Kieffer et al., 2020), GSE14764 (Denkert et al., 2009), and GSE30161 (Ferriss et al., 2012) were selected for external validation of TCGA results. Among fourteen GEO ovarian datasets available on Kaplan-Meier plotter, they were chosen for (1) cohort lager than fifty, (2) major histology component and median OS/PFS was close to TCGA OC cohort. External dataset validated ITGA3/A10/A2B/B4/B8 were significantly associated with shorter OS, ITGA6 was significantly associated with longer OS; ITGA3/B6/B8 were significantly associated with worse PFS, and ITGA6/A7 were significantly associated with better PFS (Supplementary Table 1).

In addition, we also evaluated the prognostic value of integrin family members in different clinical stages and pathological grades through Kaplan–Meier plotter. As shown in Table 2, ITGA2/A6/A7/AE/ITGB5 mRNA expression significantly correlated with better OS with HR<1 and ITGA9/ITGB3 mRNA expression significantly correlated with worse OS with HR>1 in stage I and stage II OC patients. Similarly, ITGA7/B7 mRNA expression significantly correlated with favorable OS and ITGAV/B3/B4/B6 mRNA expression significantly correlated with unfavorable OS in stage III OC; transcriptional expression of ITGA9/AV/B6 significantly correlated with longer OS, and transcriptional expression of ITGA3/A2B/B8 significantly correlated with shorter OS in OC of stage IV. Meanwhile, ITGA6 mRNA expression significantly correlated with longer OS, and ITGA2/A3/A4/A7/A10/AL/AM/B2/B6 mRNA expression significantly correlated with shorter OS in pathological grade 1 and grade 2 OC patients. ITGAE/B7 mRNA expression significantly correlated with good OS, and ITGA2B/B3/B4 mRNA expression significantly correlated with poor OS in grade 3 OC patients.

**Table 2:** The relationship between integrins and OS in different tumor grades and stages of OC (Kaplan–Meier plotter).

	**Stage I+II (42 cases)**		**Stage III (427 cases)**		**Stage IV (85 cases)**		**Grade 1+2 (75 cases)**		**Grade 3 (469 cases)**	
**Genes**	**HR (95% CI)**	***p-*value**	**HR (95% CI)**	***p-*value**	**HR (95% CI)**	***p-*value**	**HR (95% CI)**	***p-*value**	**HR (95% CI)**	***p-*value**
ITGA1	N/A	N/A	N/A	N/A	N/A	N/A	N/A	N/A	N/A	N/A
ITGA2	0.22 (0.05–0.95)	**0.03**	0.87 (0.67–1.14)	0.32	1.45 (0.78–2.7)	0.24	2.07 (1.07–4.04)	**0.03**	1.24 (0.94–1.63)	0.13
ITGA3	4.25 (0.53–34.25)	0.14	1.24 (0.95–1.61)	0.11	1.93 (1.09–3.42)	**0.02**	2.51 (1.27–4.99)	**0.01**	1.27 (0.99–1.64)	0.06
ITGA4	0.3 (0.08–1.12)	0.06	1.16 (0.89–1.51)	0.26	1.24 (0.69–2.22)	0.47	2.34 (1.17–4.67)	**0.01**	0.81 (0.6–1.09)	0.16
ITGA5	0.18 (0.02–1.44)	0.07	1.24 (0.95–1.62)	0.11	0.68 (0.36–1.27)	0.22	1.67 (0.9–3.11)	0.10	1.19 (0.92–1.54)	0.19
ITGA6	0.17 (0.04–0.8)	**0.01**	0.79 (0.61–1.03)	0.09	0.67 (0.38–1.18)	0.16	0.38 (0.19–0.76)	**4.40E-03**	0.79 (0.61–1.02)	0.07
ITGA7	0.23 (0.06–0.84)	**0.02**	0.75 (0.58–0.97)	**0.03**	1.31 (0.74–2.33)	0.36	1.97 (1.02–3.97)	**0.04**	0.74 (0.58–0.96)	**0.02**
ITGA8	3.87 (0.82–18.34)	0.07	0.81 (–0.59–1.1)	0.17	1.72 (0.94–3.16)	0.08	1.79 (0.91–3.19)	0.09	0.86 (0.64–1.15)	0.31
ITGA9	3.64 (0.97–13.6)	**0.04**	0.8 (0.6–1.07)	0.13	0.51 (0.28–0.92)	**0.02**	1.56 (0.81–3.03)	0.18	0.83 (0.65–1.08)	0.16
ITGA10	2.26 (0.46–11.05)	0.30	1.27 (0.97–1.65)	0.08	1.31 (0.74–2.35)	0.35	2.95 (1.21–7.19)	**0.01**	1.27 (0.99–1.64)	0.06
ITGA11	N/A	N/A	N/A	N/A	N/A	N/A	N/A	N/A	N/A	N/A
ITGA2B	0.56 (0.15–2.02)	0.37	1.32 (0.99–1.75)	0.06	2.69 (1.44–5)	**1.20E-03**	1.65 (0.85–3.21)	0.14	1.43 (1.08–1.91)	**0.01**
ITGAD	N/A	N/A	N/A	N/A	N/A	N/A	N/A	N/A	N/A	N/A
ITGAE	0.23 (0.06–0.87)	**0.02**	0.8 (0.62–1.05)	0.10	0.59 (0.29–1.17)	0.13	1.47 (0.79–2.72)	0.22	0.76 (0.58–0.98)	**0.04**
ITGAL	2.84 (0.76–10.64)	0.11	0.85 (0.66–1.11)	0.23	0.57 (0.3–1.1)	0.09	3.08 (1.57–6.02)	**5.90E-04**	0.81 (0.63–1.04)	0.10
ITGAM	2.28 (0.6–8.67)	0.21	1.22 (0.91–1.64)	0.18	0.77 (0.4–1.46)	0.42	2.16 (1.13–4.15)	**0.02**	0.82 (0.63–1.08)	0.16
ITGAV	0.28 (0.06–1.25)	0.08	1.36 (1.02–1.83)	**0.04**	0.49 (0.26–0.92)	**0.02**	1.56 (0.82–2.97)	0.17	1.22 (0.92–1.62)	0.17
ITGAX	0.32 (0.09–1.21)	0.08	0.86 (0.67–1.12)	0.27	0.8 (0.44–1.48)	0.48	1.54 (0.77–3.08)	0.22	0.86 (0.66–1.1)	0.23
ITGB1	0.46 (0.12–1.73)	0.24	1.13 (0.87–1.47)	0.35	1.46 (0.81–2.65)	0.20	1.78 (0.93–3.4)	0.08	1.18 (0.91–1.53)	0.20
ITGB2	0.43 (0.11–1.64)	0.21	0.76 (0.56–1.03)	0.08	1.89 (0.98–3.63)	0.05	3.2 (1.57–6.54)	**7.90E-04**	0.74 (0.55–1)	0.05
ITGB3	3.9 (1.08–14.07)	**0.03**	1.42 (1.09–1.85)	**0.01**	1.37 (0.76–2.5)	0.30	1.48 (0.72–3.04)	0.28	1.56 (1.2–2.02)	**7.90E-04**
ITGB4	4.25 (0.53–34.15)	0.14	1.37 (1.03–1.83)	**0.03**	1.58 (0.86–2.91)	0.14	1.86 (0.96–3.59)	0.06	1.35 (1.04–1.77)	**0.03**
ITGB5	0.26 (0.06–1.01)	**0.04**	1.25 (0.94–1.67)	0.13	1.52 (0.83–2.8)	0.17	0.56 (0.29–1.09)	0.08	1.3 (0.99–1.71)	0.06
ITGB6	0.25 (0.05–1.27)	0.08	1.35 (1.01–1.81)	**0.04**	0.47 (0.24–0.92)	**0.03**	1.99 (1.06–3.71)	**0.03**	1.32 (0.98–1.78)	0.07
ITGB7	2.3 (0.6–8.86)	0.21	0.72 (0.53–0.98)	**0.04**	1.55 (0.83–2.89)	0.16	0.63 (0.33–1.2)	0.15	0.67 (0.49–0.92)	**0.01**
ITGB8	0.42 (0.11–1.61)	0.19	1.25 (0.95–1.65)	0.11	1.92 (1.06–3.48)	**0.03**	1.65 (0.87–3.12)	0.12	1.29 (0.98–1.71)	0.07

*Bold font indicates significant difference*.

With respect to PFS (Table 3), mRNA expression of ITGA6/A10/AE significantly correlated with longer PFS, and ITGA3/A4/AL/B2 significantly correlated with shorter PFS in stage I and stage II OC patients; ITGA4/A6/A7/B1 significantly correlated with good PFS, ITGB4 significantly correlated with poor PFS in stage III OC patients; ITGA2/A4/A6/AV/B6 significantly correlated with favorable PFS, ITGA3/B3/B4 significantly correlated with unfavorable PFS in stage IV OC patients. With regard to pathological grades, ITGA6/A9/B1/B7 significantly correlated with longer PFS, ITGA2/AL/B2 significantly correlated with shorter PFS in grade 1 and grade 2 OC patients; ITGA4A6/A7/AX/B1 significantly correlated with better PFS, ITGA3/AM/B4/B8 significantly correlated with worse PFS in grade 3 OC patients.

**Table 3:** The relationship between integrins and PFS in different tumor grades and stages of OC (Kaplan–Meier plotter).

	**Stage I+II (39 cases)**		**Stage III (400 cases)**		**Stage IV (81 cases)**		**Grade 1+2 (71 cases)**		**Grade 3 (44 cases)**	
**Genes**	**HR (95% CI)**	***p-*value**	**HR (95% CI)**	***p-*value**	**HR (95% CI)**	***p-*value**	**HR (95% CI)**	***p-*value**	**HR (95% CI)**	***p-*value**
ITGA1	N/A	N/A	N/A	N/A	N/A	N/A	N/A	N/A	N/A	N/A
ITGA2	3.46 (0.78–15.36)	0.08	0.87 (0.67–1.12)	0.28	0.53 (0.28–0.99)	**0.04**	1.82 (1–3.32)	**0.05**	0.88 (0.68–1.14)	0.33
ITGA3	5.32 (1.68–16.84)	**1.50E-03**	0.79 (0.6–1.03)	0.08	2.12 (1.13–3.97)	**0.02**	1.31 (0.73–2.36)	0.37	1.3 (1–1.69)	**0.05**
ITGA4	4.65 (1.46–14.87)	**0.01**	0.71 (0.54–0.94)	**0.01**	0.38 (0.2–0.74)	**2.80E-03**	1.85 (0.98–3.51)	0.06	0.67 (0.52–0.88)	**3.90E-03**
ITGA5	0.51 (0.14–1.82)	0.29	1.11 (0.85–1.47)	0.44	1.53 (0.75–3.1)	0.24	0.6 (0.29–1.26)	0.18	1.24 (0.94–1.63)	0.13
ITGA6	0.34 (0.11–1.03)	**0.05**	0.71 (0.55–0.91)	**0.01**	0.47 (0.25–0.9)	**0.02**	0.4 (0.22–0.72)	**1.70E-03**	0.68 (0.53–0.88)	**3.50E-03**
ITGA7	3.97 (0.52–30.32)	0.15	0.57(0.44–0.74)	**1.60E-05**	1.71 (0.92–3.16)	0.09	0.59 (0.32–1.11)	0.10	0.69 (0.54–0.88)	**3.40E-03**
ITGA8	2.95 (0.93–9.3)	0.05	0.8 (0.61–1.04)	0.10	0.69 (0.37–1.29)	0.24	0.61 (0.33–1.12)	0.11	1.24 (0.85–1.48)	0.42
ITGA9	2.15 (0.76–6.1)	0.14	0.77 (0.58–1.02)	0.07	0.68 (0.35–1.34)	0.26	0.34 (0.15–0.77)	**0.01**	1.2 (0.91–1.57)	0.19
ITGA10	0.27 (0.08–0.84)	**0.02**	0.79 (0.61–1.04)	0.10	0.57 (0.31–1.04)	0.06	1.36 (0.74–2.47)	0.32	0.8 (0.61–1.03)	0.09
ITGA11	N/A	N/A	N/A	N/A	N/A	N/A	N/A	N/A	N/A	N/A
ITGA2B	0.61 (0.21–1.8)	0.37	1.27 (0.95–1.69)	0.11	1.27 (0.64–2.55)	0.49	0.72 (0.38–1.35)	0.30	1.31 (0.99–1.72)	0.06
ITGAD	N/A	N/A	N/A	N/A	N/A	N/A	N/A	N/A	N/A	N/A
ITGAE	0.35 (0.13–0.99)	**0.04**	1.24 (0.94–1.63)	0.13	0.57 (0.28–1.16)	0.12	0.61 (0.34–1.1)	0.10	1.26 (0.97–1.63)	0.08
ITGAL	8.12 (1.06–62.35)	**0.02**	0.87 (0.67–1.14)	0.32	0.58 (0.29–1.13)	0.11	2.25 (1.23–4.12)	**0.01**	0.88 (0.68–1.13)	0.31
ITGAM	1.91 (0.68–5.36)	0.21	1.31 (0.99–1.74)	0.06	0.62 (0.34–1.13)	0.12	0.6 (0.34–1.09)	0.09	1.36 (1.04–1.78)	**0.03**
ITGAV	0.62 (0.21–1.83)	0.39	1.16 (0.89–1.5)	0.27	0.4 (0.2–0.8)	**0.01**	1.6 (0.89–2.9)	0.11	0.83 (0.65–1.07)	0.16
ITGAX	1.53 (0.54–4.33)	0.42	0.81 (0.63–1.05)	0.11	0.5 (0.23–1.09)	0.08	0.57 (0.27–1.19)	0.13	0.78 (0.6–1)	**0.05**
ITGB1	0.25 (0.06–1.13)	0.05	0.67 (0.5–0.88)	**3.70E−03**	1.58 (0.7–3.58)	0.26	0.52 (0.28–0.98)	**0.04**	0.75 (0.58–0.97)	**0.03**
ITGB2	4.24 (0.95–18.91)	**0.04**	0.82 (0.62–1.08)	0.16	1.67 (0.87–3.32)	0.12	2.2 (1.19–4.08)	**0.01**	0.87 (0.66–1.14)	0.31
ITGB3	2.12 (0.75–6)	0.15	0.83 (0.64–1.08)	0.17	1.94 (0.99–3.78)	**0.05**	1.41 (0.78–2.53)	0.25	1.24 (0.95–1.62)	0.11
ITGB4	0.53 (0.18–1.57)	0.25	1.32 (1.01–1.73)	**0.04**	2.07 (1.09–3.94)	**0.02**	1.51 (0.79–2.91)	0.21	1.44 (1.11–1.87)	**0.01**
ITGB5	0.65 (0.23–1.84)	0.41	0.78 (0.6–1)	0.05	1.69 (0.91–3.13)	0.09	1.33 (0.71–2.51)	0.37	0.8 (0.62–1.03)	0.09
ITGB6	0.63 (0.22–1.82)	0.39	1.22 (0.92–1.63)	0.17	0.43 (0.2–0.94)	**0.03**	1.37 (0.76–2.46)	0.30	1.31 (0.99–1.74)	0.06
ITGB7	2.45 (0.81–7.45)	0.10	0.77 (0.58–1)	0.05	1.51 (0.83–2.75)	0.17	0.51 (0.28–0.93)	**0.03**	0.84 (0.63–1.12)	0.23
ITGB8	1.94 (0.68–5.52)	0.21	1.29 (0.97–1.71)	0.08	1.62 (0.86–3.05)	0.13	1.84 (0.97–3.49)	0.06	1.33 (1–1.76)	**0.05**

*Bold font indicates significant difference*.

Taken together, these results indicated that ITGA6 was a potential good prognosis factor in OC, especially valuable for the OS prediction in OC patients diagnosed with early stage or low grade, and valuable for PFS prediction in OC patients with all stages or grades. Our study also supports that ITGA3/ B4 might serve as primary poor prognosis factors in OC across multiple stages and grades; while ITGB8 mainly valuable for poor prognosis of advanced stage OS and high grade PFS in OC. Complementary, a set of integrins included favorable ITGAE/B7 and unfavorable ITGAL/AM/B2, mainly focused on early stage or grade OC predictions. However, these stage/grade-specific prognostic factors may need further validation.

### Association With Clinical Parameters and Prognostic Independence of Integrin Genes

Next, we retrieved the clinical data and mRNA expression of TCGA ovarian cancer from the GDC data portal. There were 375 cases available with files of mRNA expression and matched clinical data. This set was used to analyze the association of particular integrin genes with clinical parameters and the Cox regression analysis. As primary prognostic factors for both OS and PFS, ITGA3/A6/B4/B8 were further analyzed. As indicated in the Supplementary Table 2, we found ITGA3 significantly associated with primary therapy outcome (*p* < 0.0001), ITGA6 significantly associated with OS status (*p* = 0.026), ITGB4 significantly associated with cancer status (*p* = 0.042) and tumor residual (*p* = 0.024), ITGB8 significantly associated with chemotherapy (*p* = 0.020). No significant association of specific integrin genes with age at diagnosed, race, ethnicity, stage, grade, PFS status, hormone therapy, immune therapy, and target therapy had been found.

Nevertheless, in the following univariant cox regression analysis, clinical features including chemotherapy, cancer status, tumor residual, primary therapy outcome were significantly associated with OS and PFS; while age at diagnosed, race only significantly associated with OS, and ethnicity only significantly associated with PFS. Meantime, high expression of ITGA3/B4/B8 were also significantly associated with poor OS, and high expression of ITGB4/B8 significantly associated with poor PFS (Table 4). However, clinical features including race, ethnicity, grade, cancer status, tumor residual, primary therapy outcome were not included for further multivariant cox regression analysis, due to considerable records missing. Multivariant cox regression analysis showed high expression of B4/B8 were independently poor prognostic factors of both OS and PFS, and high expression of ITGA3 were independently poor prognostic factor of OS in OC (Table 5).

**Table 4:** Univariant Cox regression analysis of OS and PFS in OC patients.

**Variables**	**OS Univariant Cox**			**PFS Univariant Cox**		
	**HR**	**95% CI**	***p*-value**	**HR**	**95% CI**	***p*-value**
Age (<60/≥60)	1.315	1.014–1.706	**0.039**	1.171	0.9251–1.483	0.189
Race (White/Others)	1.691	1.093–2.617	**0.0183**	1.194	0.8058–1.77	0.376
Ethnicity (Non-Hispanic or Latinic/Hispanic or Latinic)	1.018	0.4159–2.494	0.968	2.243	1.047–4.803	**0.0376**
Grade (G1+G2/G3+G4)	1.213	0.81–1.818	0.348	1.205	0.8401–1.728	0.311
Stage (I+II/III+IV)	2.007	0.8908–4.523	0.0928	1.652	0.926–2.948	0.0892
Chemotherapy (NO/YES)	0.3621	0.2317–0.5658	**8.17E-06**	0.6455	0.4205–0.9908	**4.52E-03**
Hormonetherapy (NO/YES)	0.9375	0.6268–1.402	0.7	1.058	0.7354–1.522	0.761
Immunotherapy (NO/YES)	0.683	0.3028–1.54	0.358	0.9425	0.4847–1.833	0.681
Targettherapy (NO/YES)	0.6409	0.3906–1.051	0.07	1.024	0.673–1.557	0.913
Cancer_status (Tumor free/With tumor)	8.743	4.74–16.13	**3.87E-12**	9.997	6.013–16.62	** <2E-16**
Tumor_residual (No macroscopic/Macroscopic)	2.154	1.384–3.352	**0.000676**	1.862	1.318–2.633	**0.000429**
Primary_Therapy_Outcome (Response/non-Response)	3.252	2.204–4.798	**2.81E-09**	2.463	1.749–3.468	**2.45E-07**
ITGA3 (Low/High)	1.444	1.077–1.937	**0.0141**	1.187	0.908–1.55	0.21
ITGA6 (Low/High)	0.8175	0.6063–1.102	0.186	0.8574	0.6772–1.085	0.201
ITGB4 (Low/High)	1.346	1.004–1.803	**0.0467**	1.357	1.07–1.72	**0.0117**
ITGB8 (Low/High)	1.531	1.15–2.039	**0.00356**	1.397	1.1–1.773	**0.00603**

*Bold font indicates significant difference*.

**Table 5:** Multivariant Cox regression analysis of OS and PFS in OC patients.

**Variables**	**ITGA3**			**ITGA6**			**ITGB4**			**ITGB8**		
	**HR**	**95% CI**	***p*-value**	**HR**	**95% CI**	***p*-value**	**HR**	**95% CI**	***p*-value**	**HR**	**95% CI**	***p*-value**
**A.OS**												
Age (<60/≥60)	1.333	1.0240–1.7352	**0.032**	1.3028	0.9972–1.7022	0.0525	1.3578	1.0429–1.7676	**0.0231**	1.3819	1.0597–1.8021	**0.01693**
Stage (I+II/III+IV)	2.1172	0.9329–4.8045	0.0728	1.8666	0.8231–4.2330	0.1352	2.0041	0.8846–4.5407	0.0957	2.1571	0.9475–4.9113	0.06704
Chemotherapy (NO/YES)	0.3846	0.2442–0.6058	**3.74E-05**	0.3596	0.2287–0.5655	**9.51E-06**	0.3732	0.2375–0.5864	**1.92E-05**	0.3942	0.2494–0.6229	**6.68E-05**
Hormonetherapy (NO/YES)	1.0106	0.6712–1.5217	0.9596	0.9792	0.6504–1.4743	0.92	1.0144	0.6737–1.5275	0.9453	1.0247	0.6809–1.5421	0.90683
Immunotherapy (NO/YES)	0.8538	0.3736–1.9514	0.7078	0.8208	0.3587–1.8782	0.6402	0.8324	0.3643–1.9021	0.6635	0.8694	0.3804–1.9868	0.73994
Targettherapy (NO/YES)	0.6727	0.4072–1.1114	0.1217	0.7017	0.4253–1.1579	0.1656	0.6868	0.4160–1.1337	0.1418	0.7335	0.4448–1.2095	0.2245
Indicated Gene (Low/High)	1.4733	1.0964–1.9797	**0.0102**	0.8145	0.6001–1.1056	0.1882	1.4038	1.0462–1.8837	**0.0238**	1.5314	1.1440–2.0499	**0.00418**
**B.PFS**												
Age (<60/≥60)	1.167	0.9191–1.483	0.2047	1.1582	0.9095–1.475	0.2337	1.2134	0.9546–1.542	0.114	1.1842	0.9328–1.503	0.16494
Stage (I+II/III+IV)	1.674	0.9293–3.0160	0.0862	1.5833	0.8783–2.854	0.1264	1.7056	0.9487–3.067	0.0744	1.7396	0.9650–3.136	0.06555
Chemotherapy (NO/YES)	0.667	0.4320–1.030	0.0676	0.652	0.4221–1.007	0.0538	0.7154	0.4613–1.109	0.1346	0.7299	0.4699–1.134	0.16125
Hormonetherapy (NO/YES)	1.047	0.7240–1.515	0.8056	1.0348	0.7150–1.498	0.856	1.0166	0.7024–1.471	0.9305	1.0423	0.7202–1.508	0.82596
Immunotherapy (NO/YES)	1.109	0.5637–2.182	0.7644	1.0937	0.5563–2.150	0.795	1.1132	0.5664–2.188	0.7557	0.9874	0.4996–1.952	0.97098
Targettherapy (NO/YES)	1.044	0.6825–1.598	0.8414	1.058	0.6915–1.619	0.7951	0.9993	0.6518–1.532	0.9975	1.1014	0.7193–1.687	0.65678
Indicated Gene (Low/High)	1.191	0.9102–1.559	0.2026	0.8831	0.6949–1.122	0.3095	1.3724	1.0777–1.748	**0.0103**	1.4117	1.1048–1.804	**0.00583**

*Bold font indicates significant difference*.

### Association of Integrin Genes With Ovarian Cancer Metastasis and Drug-Resistance

**Figure 4:**
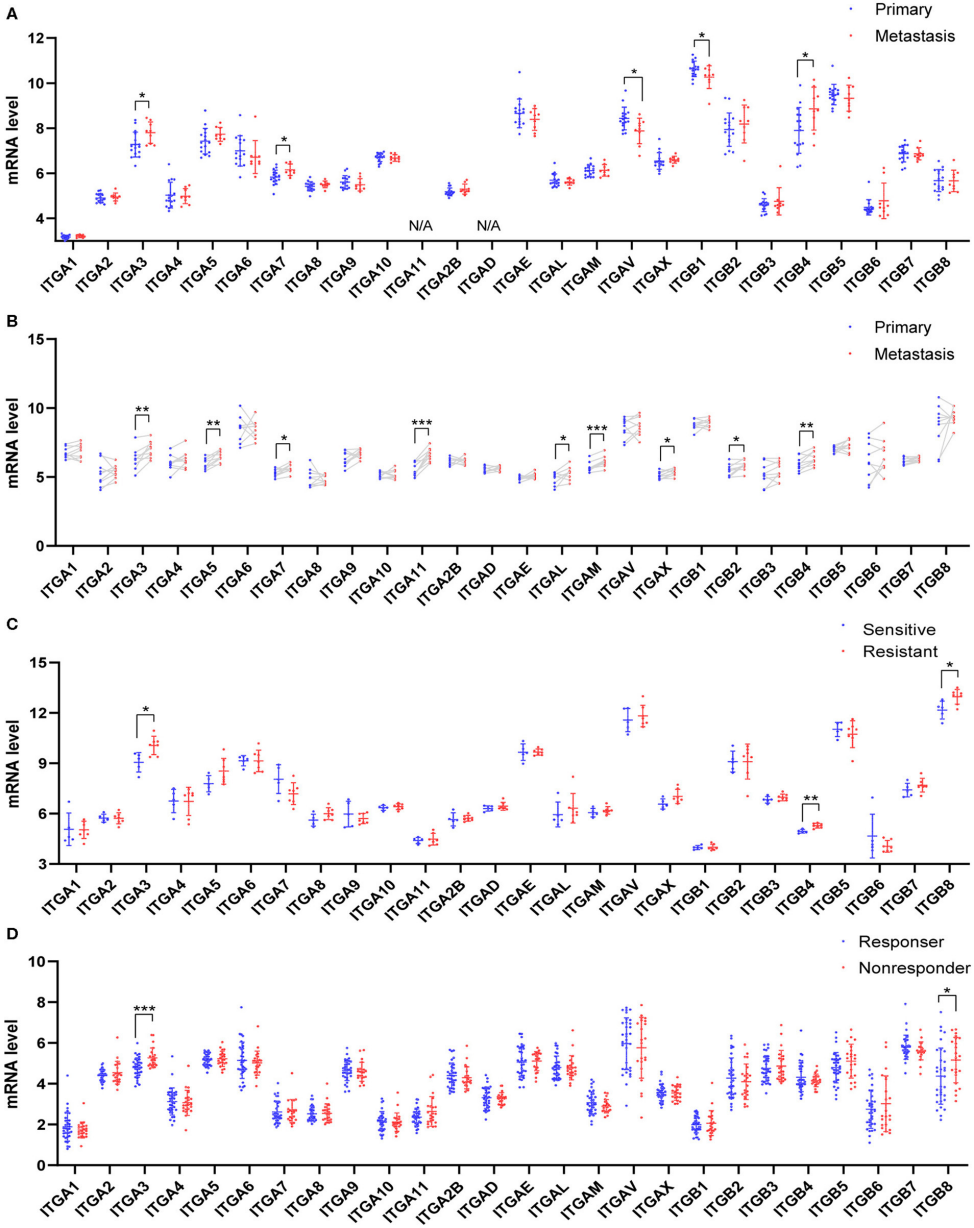
Relationship between integrin genes mRNA expression and metastasis or platinum resistance in OC. **(A)** Integrin genes transcriptional expression in unpaired primary and metastatic OC tissues, number (primary) = 16, number (metastasis) = 9. **(B)** Integrin genes transcriptional expression in paired primary and metastatic OC tissues, number (primary) = 9, number (metastasis) = 9. **(C)** Integrin genes transcriptional expression in platinum-sensitive and platinum-resistant OC tissues, number (sensitive) = 5, number (resistant) = 7. **(D)** Integrin genes transcriptional expression in platinum-based chemotherapy responder and non-responders OC tissue, number (responder) = 33, number (non-responder) = 22. *p < 0.05, **p < 0.01, ***p < 0.001.

Due to the difficulty in early diagnosis, about 2/3 ovarian cancer patients diagnosed at a late stage with extensive peritoneum metastasis, made the ovarian cancer so-called “silent killer.” Integrin members played an important role in cell-matrix attachment in normal epithelial cells. It was possible some of them participated in the ovarian cancer metastasis process, such as colonization at distal. Here, we analyzed two distinct GEO datasets which compared the metastasis ovarian cancer to primary ovarian cancer. In the GSE131978 (Tassi et al., 2019) dataset, a group of primary OC samples were compared to a group of omental metastasis OC samples. The result showed ITGA3/7 and ITGB4 were significantly higher expressed in metastasis OC samples, ITGAV and ITGB1 were significantly higher expressed in primary OC samples (Figure 4A). Furthermore, the GSE30587 (Brodsky et al., 2014) dataset compared the primary OC samples and metastasis OC samples from the same patients. Paired *t*-test analysis showed the mRNA expression of ITGA3/A5/A7/A11/AL/AM/AX and ITGB2/B4 were significantly higher in the metastasis OC samples (Figure 4B). Combinatory, ITGA3/A7 and ITGB4 might be important for OC metastasis.

Most OC patients received surgery and chemotherapy after the initial diagnosis. The standard first-line chemotherapy was platinum-based chemotherapy. Platinum response was important for the prognosis of OC patients. The platinum-resistant generally led to a poor outcome and remains a major impediment in the treatment. To explore whether integrin genes were associated with intrinsic chemotherapy resistance, we retrieved the transcription data from GEO with ovarian cancer chemotherapy response study. The GSE131978 (Tassi et al., 2019) defined patients with tumor progression time <6 months as relatively resistant, and patients with tumor progression time more than 12 months as relative sensitive. The analysis showed ITGA3 and ITGB4/8 significantly overexpressed in the platinum-resistant group (Figure 4C). Another GEO dataset GSE30161 (Ferriss et al., 2012), compared to the responders and non-responders post-platinum-based chemotherapy, showed ITGA3/B8 were significantly higher in non-responders (Figure 4D). However, in the GSE51373 (Koti et al., 2013) which compared resistant patients with <8 months PFS to sensitive patients with more than 18 months PFS, showed no significant difference in integrins expression (data didn't show). These results indicated ITGA3 and ITGB8 might be important for platinum-resistance.

### Correlation Between Integrin Genes Expression and the Infiltration of Immune Cells in Ovarian Cancer

Despite the traditional surgery and chemotherapy, immunotherapy is a novel and promising treatment strategy. Ovarian cancer is immunogenic, about 50% of patients had spontaneous tumor immune responses with an abundance of immune cell infiltration. T cell-rich ovarian cancer was associated with improved OS and PFS (Zhang et al., 2003; Goode et al., 2017; Garsed et al., 2018; Marth et al., 2019), it indicated that stronger antitumor immune response might mean longer survival of OC patients. Meanwhile, T cell exhaustion and markers like PD-L1 might affect immune therapy efficacy (Abiko et al., 2013; Duraiswamy et al., 2013; Webb et al., 2015). Therefore, we investigate the relationship between integrin genes and immune infiltration levels in OC from TIMER.

**Figure 5:**
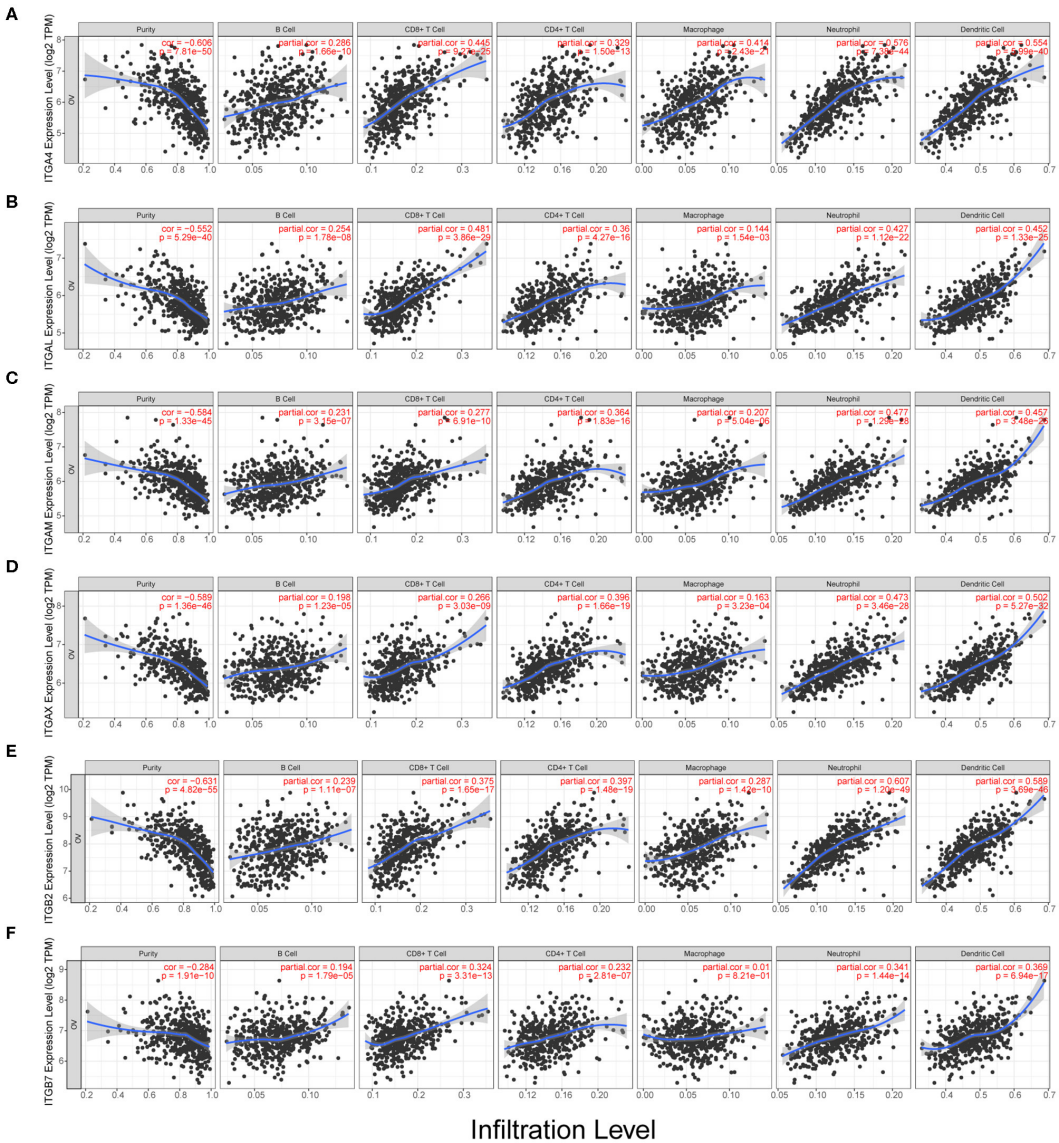
Correlation analysis of integrin genes and infiltration levels of immune cells in OC tissues. **(A)** Correlation of ITGA4 mRNA and infiltration levels. **(B)** Correlation of ITGAL mRNA and infiltration levels. **(C)** Correlation of ITGAM mRNA and infiltration levels. **(D)** Correlation of ITGAX mRNA and infiltration levels. **(E)** Correlation of ITGB2 mRNA and infiltration levels. **(F)** Correlation of ITGB7 mRNA and infiltration levels.

We observed the ITGA1/A2/A4/A5/A7/A8/A11/A2B/AL/ AM/AV/AX and ITGB1/B2/B3/B5/B6/B7 showed significantly obvious correlation with tumor purity in OC. Among them, ITGB2 showed highest correlation ratio (*r* = −0.63). Detail correlation between immune cell type and each integrin member were showed in Figure 5 and Supplementary Figure 2. In specific, ITGA1 significantly correlated with macrophage (*r* = 0.23); ITGA4 showed obvious correlation with neutrophil (*r* = 0.58) and dendritic cell (*r* = 0.55), CD8+ T cell (*r* = 0.45), macrophage (*r* = 0.41), CD4+ T cell (*r* = 0.33), B cell (*r* = 0.29); ITGAE significantly correlated with CD8+ T cell (*r* = 0.24); ITGAL showed significant correlation with CD8+ T cell (*r* = 0.48), dendritic cell (*r* = 0.45), neutrophil (*r* = 0.43), CD4+ T cell (*r* = 0.36) and B cell (*r* = 0.25); ITGAM showed significant correlation with neutrophil (*r* = 0.48), dendritic cell (*r* = 0.46), CD4+ T cell (*r* = 0.36), CD8+ T cell (*r* = 0.28), B cell (*r* = 0.23) and macrophage (*r* = 0.21); ITGAX showed significant correlation with dendritic cell (*r* = 0.50), neutrophil (*r* = 0.47), CD4+ T cell (*r* = 0.40), CD8+ T cell (*r* = 0.27) and B cell (*r* = 0.20); ITGB1 significantly correlated with B cell (*r* = −0.2); ITGB2 showed significant correlation with neutrophil (*r* = 0.61), dendritic cell (*r* = 0.59), CD4+ T cell (*r* = 0.4), CD8+ T cell (*r* = 0.38), macrophage (*r* = 0.29), B cell (*r* = 0.24); ITGB4 significantly correlated with macrophage (*r* = −0.2); ITGB6 significantly correlated with neutrophil (*r* = 0.26); ITGB7 significantly correlated with dendritic cell (*r* = 0.37), neutrophil (*r* = 0.34), CD8+ T cell (*r* = 0.32) and CD4+ T cell (*r* = 0.23). While ITGA8/A9/A10/A11 and ITGB3/B5/B8 didn't show significant correlation with any specific immune cell type in TIMER database. In conclusion, ITGA4/AL/AM/AX and ITGB2/B7 might have multiple and closely function in immune infiltration. Specifically, ITGA4/AL/AM/AX and ITGB2 were more closely associated with neutrophil and dendritic cell infiltration; ITGA4/AL were more closely associated with CD8+ T cell infiltration; while ITGAX and ITGB2 were more closely associated with CD4+ T cell infiltration.

### mRNA Correlation Analysis Between Integrin Genes and Tumor Infiltrate Markers

To further explore the correlation between integrin gens and immune infiltrates in ovarian cancer, we analyzed the mRNA correlation between integrin genes and markers of diverse immune infiltrates by TIMER database. Typical markers of CD8+ T cells, T cells (general), B cells, monocytes, TAMs, M1 and M2 macrophage, neutrophils, NK cells, DCs, Th1 cells, Th2 cells, Tfh cells, Th17 cells, Tregs and exhausted T cells reported by others were included (Sousa and Määttä, 2016; Danaher et al., 2017; Siemers et al., 2017).

After adjustment by tumor purity (Yoshihara et al., 2013), we found ITGA1/A5/A8/A11/AD/AV and ITGB1 showed weakly correlation with macrophage markers in OC, ITGA5/A11 and ITGB5 weakly correlated with Treg markers, ITGAD and ITGB6 showed weakly correlation with natural killer cells markers. ITGA2/A3/A6/A7/A9/A10/A2B/AE and ITGB3/B4 only showed weak correlations with sporadic markers (Data not shown).

We observed prominent correlation in ITGA4/AL/AM/AX and ITGB2/B7 with majority of TIL markers in OC, except B cell, M1 macrophage and natural killer cell markers (Table 6). Among them, ITGA4/AL/AM/AX and ITGB2/B7 showed especially high correlation with monocyte, M2 macrophage and T cell exhaustion markers. ITGA4/AL/AM/AX and ITGB2/B7 showed relatively moderate correlation with T cell general, CD8+ T cell, neutrophil, dendritic cell Th1 and Treg markers. ITGA4/AL/AM/AX and ITGB2/B7 also showed weak correlation with Th2 and Tfh markers, and less correlation with M1 macrophage, Th17, B cell, natural killer cell markers.

**Table 6:** Correlation analysis between integrin genes and markers of immune cells.

**Description**	**Gene markers**	**ITGA4**		**ITGAL**		**ITGAM**		**ITGAX**		**ITGB2**		**ITGA7**	
		**cor**	***p***	**cor**	***p***	**cor**	***p***	**cor**	***p***	**cor**	***p***	**cor**	***p***
CD8+ T cell	CD8A	0.43	^****^	0.65	^****^	0.31	^****^	0.42	^****^	0.41	^****^	0.36	^****^
	CD8B	0.29	^****^	0.45	^****^	0.17	^**^	0.27	^****^	0.24	^***^	0.27	^****^
T cell (general)	CD3D	0.37	^****^	0.67	^****^	0.31	^****^	0.41	^****^	0.45	^****^	0.41	^****^
	CD3E	0.45	^****^	0.79	^****^	0.39	^****^	0.52	^****^	0.51	^****^	0.50	^****^
	CD2	0.50	^****^	0.79	^****^	0.43	^****^	0.55	^****^	0.54	^****^	0.50	^****^
B cell	CD19	0.08	0.18	0.25	^****^	0.05	0.41	0.10	0.12	0.02	0.75	0.26	^****^
	CD79A	0.23	^***^	0.42	^****^	0.17	^**^	0.22	^***^	0.19	^**^	0.26	^****^
Monocyte	CD86	0.74	^****^	0.81	^****^	0.81	^****^	0.80	^****^	0.82	^****^	0.50	^****^
	CSF1R	0.75	^****^	0.74	^****^	0.87	^****^	0.80	^****^	0.88	^****^	0.43	^****^
TAM	CCL2	0.30	^****^	0.35	^****^	0.39	^****^	0.41	^****^	0.43	^****^	0.24	^***^
	CD68	0.69	^****^	0.75	^****^	0.83	^****^	0.80	^****^	0.83	^****^	0.52	^****^
	IL10	0.42	^****^	0.31	^****^	0.35	^****^	0.30	^****^	0.37	^****^	0.12	0.06
M1 Macrophage	NOS2	0.11	0.09	−0.07	0.26	0.00	0.96	−0.04	0.57	−0.05	0.46	−0.09	0.16
	IRF5	0.33	^****^	0.40	^****^	0.42	^****^	0.41	^****^	0.44	^****^	0.38	^****^
	PTGS2	0.14	^*^	−0.03	0.62	0.12	0.05	0.08	0.19	0.04	0.49	−0.05	0.42
M2 Macrophage	CD163	0.70	^****^	0.70	^****^	0.81	^****^	0.72	^****^	0.78	^****^	0.40	^****^
	VSIG4	0.63	^****^	0.57	^****^	0.72	^****^	0.58	^****^	0.70	^****^	0.28	^****^
	MS4A4A	0.68	^****^	0.67	^****^	0.67	^****^	0.61	^****^	0.68	^****^	0.32	^****^
Neutrophils	CEACAM8	0.06	0.35	0.03	0.60	0.07	0.29	0.09	0.14	0.00	0.97	0.04	0.54
	ITGAM	0.69	^****^	0.68	^****^	−1.00	^****^	0.83	^****^	0.85	^****^	0.48	^****^
	CCR7	0.43	^****^	0.66	^****^	0.39	^****^	0.46	^****^	0.46	^****^	0.38	^****^
Natural killer cell	KIR2DL1	0.11	0.09	0.16	^*^	0.08	0.22	0.12	0.05	0.12	0.06	0.19	^**^
	KIR2DL3	0.12	0.05	0.20	^**^	0.21	^***^	0.22	^***^	0.25	^****^	0.25	^****^
	KIR2DL4	0.29	^****^	0.51	^****^	0.29	^****^	0.38	^****^	0.36	^****^	0.38	^****^
	KIR3DL1	0.12	0.06	0.33	^****^	0.18	^**^	0.26	^****^	0.19	^**^	0.28	^****^
	KIR3DL2	0.14	^*^	0.16	^*^	0.07	0.24	0.14	^*^	0.13	^*^	0.06	0.36
	KIR3DL3	−0.06	0.37	0.08	0.20	0.06	0.37	0.08	0.19	0.05	0.46	0.07	0.30
	KIR2DS4	0.07	0.30	0.19	^**^	0.10	0.11	0.16	^**^	0.12	0.07	0.16	^**^
Dendritic cell	HLA-DPB1	0.45	^****^	0.63	^****^	0.54	^****^	0.57	^****^	0.62	^****^	0.45	^****^
	HLA-DQB1	0.24	^***^	0.41	^****^	0.31	^****^	0.38	^****^	0.35	^****^	0.29	^****^
	HLA-DRA	0.44	^****^	0.55	^****^	0.50	^****^	0.50	^****^	0.57	^****^	0.38	^****^
	HLA-DPA1	0.48	^****^	0.65	^****^	0.55	^****^	0.57	^****^	0.61	^****^	0.45	^****^
	CD1C	0.24	^***^	0.30	^****^	0.41	^****^	0.38	^****^	0.48	^****^	0.22	^***^
	NRP1	0.37	^****^	0.24	^***^	0.37	^****^	0.33	^****^	0.32	^****^	0.08	0.21
	ITGAX	0.67	^****^	0.76	^****^	0.83	^****^	−1.00	^****^	0.82	^****^	0.60	^****^
Th1	TBX21	0.45	^****^	0.80	^****^	0.43	^****^	0.54	^****^	0.51	^****^	0.56	^****^
	STAT4	0.52	^****^	0.67	^****^	0.41	^****^	0.51	^****^	0.42	^****^	0.38	^****^
	STAT1	0.35	^****^	0.49	^****^	0.28	^****^	0.33	^****^	0.21	^***^	0.35	^****^
	IFNG	0.35	^****^	0.61	^****^	0.23	^***^	0.36	^****^	0.32	^****^	0.41	^****^
	TNF	0.13	^*^	0.17	^**^	0.29	^****^	0.38	^****^	0.31	^****^	0.36	^****^
Th2	GATA3	0.24	^***^	0.22	^***^	0.19	^**^	0.24	^***^	0.21	^***^	0.12	0.07
	STAT6	0.25	^****^	0.25	^****^	0.33	^****^	0.30	^****^	0.28	^****^	0.30	^****^
	STAT5A	0.36	^****^	0.42	^****^	0.51	^****^	0.47	^****^	0.48	^****^	0.37	^****^
	IL13	0.11	0.08	0.14	^*^	0.13	^*^	0.21	^***^	0.14	^*^	0.14	^*^
Tfh	BCL6	0.24	^***^	0.29	^****^	0.28	^****^	0.33	^****^	0.30	^****^	0.23	^***^
	IL21	0.23	^***^	0.32	^****^	0.17	^**^	0.24	^***^	0.22	^***^	0.28	^****^
Th17	STAT3	0.40	^****^	0.35	^****^	0.42	^****^	0.41	^****^	0.35	^****^	0.23	^***^
	IL17A	0.08	0.21	0.08	0.23	0.08	0.20	0.06	0.36	0.11	0.09	0.08	0.21
Treg	FOXP3	0.43	^****^	0.68	^****^	0.43	^****^	0.58	^****^	0.45	^****^	0.50	^****^
	CCR8	0.34	^****^	0.45	^****^	0.41	^****^	0.45	^****^	0.39	^****^	0.34	^****^
	STAT5B	0.31	^****^	0.20	^**^	0.28	^****^	0.25	^****^	0.18	^**^	0.09	0.18
	TGFB1	0.50	^****^	0.42	^****^	0.48	^****^	0.48	^****^	0.50	^****^	0.27	^****^
T cell exhaustion	PDCD1	0.38	^****^	0.60	^****^	0.30	^****^	0.42	^****^	0.42	^****^	0.39	^****^
	CTLA4	0.45	^****^	0.70	^****^	0.40	^****^	0.52	^****^	0.45	^****^	0.44	^****^
	LAG3	0.45	^****^	0.69	^****^	0.39	^****^	0.49	^****^	0.41	^****^	0.49	^****^
	HAVCR2	0.74	^****^	0.77	^****^	0.82	^****^	0.81	^****^	0.84	^****^	0.49	^****^
	GZMB	0.22	^***^	0.52	^****^	0.18	^*^	0.29	^****^	0.30	^****^	0.37	^****^

* *p < 0.05*,

** *p < 0.01*,

*** *p < 0.001*,

**** *p < 0.0001*.

Furthermore, we noticed the integrin transcriptional expression data from TCGA RNA seq, automatically clustered into 4 groups. One of the groups contained all the TIL highly associated integrin members, ITGA4/AL/AM/AX/B2/B7, which lacked of significantly association with drug resistance or metastasis, but major of them showed significantly associated with prognosis in early stage or grade. Another group contained integrin members ITGA3/B4/B8, which were significantly associated with drug resistance, metastasis and unfavored prognosis in advanced stage or grade (Supplementary Figure 3A). These results indicate the existence of TIL associated integrins in OC.

Studies proved malignant cancer cells and other cells recruit monocyte to OC microenvironment and differentiate into TAM (tumor-associated macrophage; Gupta et al., 2018). Most of macrophage and monocyte polarize into M2 macrophage and promote tumor progression and suppress immune response (Zhang et al., 2014; Carroll et al., 2016). Exhausted T cells might associate with poor prognosis by immunosuppressive mechanism and limiting antitumor response (Abiko et al., 2013; Webb et al., 2015; Alsaab et al., 2017). Thus, ITGA4/AL/AM/AX and ITGB2/7 might also render a promoting role in OC. Nonetheless, it's worth noting that some of the integrins might express on both immune cells and epithelial cells, unraveling the complicated interaction between TIL and integrins in OC still need further research.

### Possible Mechanisms of ITGA3 and ITGB4 Involved in Ovarian Cancer

**Figure 6:**
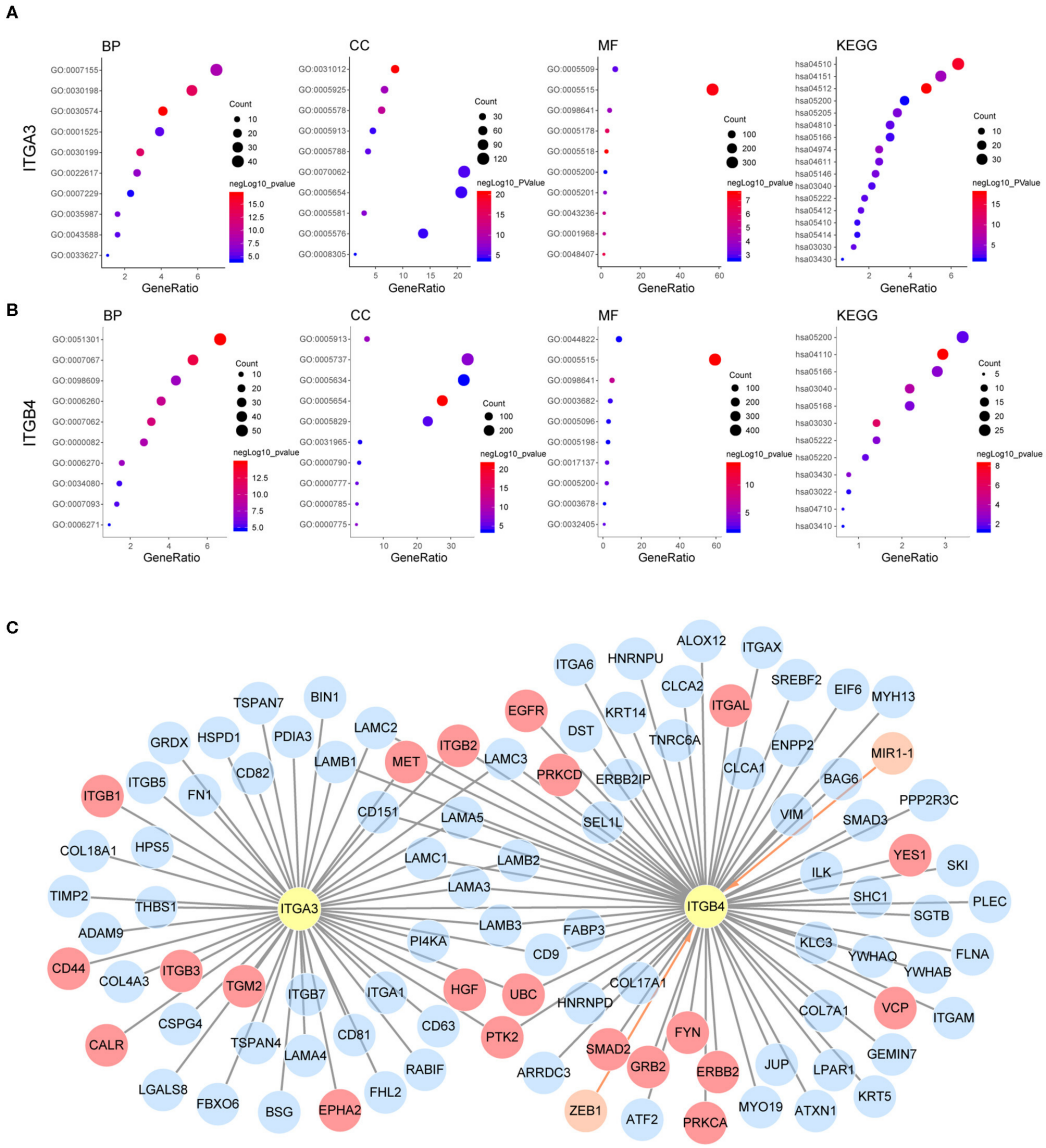
Possible mechanism of ITGA3 and ITGB4 involved in OC. Top10 significantly enriched GO TERM and all significant enriched KEGG pathways for **(A)** ITGA3 and **(B)** ITGB4. **(C)** Regulatory network of ITGA3 and ITGB4, nodes in orange and edges with an arrow represents upstream transcriptional regulator, nodes with gray edges showed partners with physical protein interaction, gray edges with red nodes showed interaction partners with available drug basing on drugbank database.

Our previous analysis revealed ITGA3/B4/B8 were associated with both poor OS and PFS, their prognostic role in OS were confirmed by external GEO datasets and independent as revealed by multivariant cox regression analysis. Other integrins like ITGA5/A2B/B3 were potential risk factors for OS, ITGB6 was a potential risk factor for PFS, ITGAE/B7 were potential protective factors for OS, ITGA4/AX/B1 were potential protective factors for PFS, and ITGA6/A7 were protective factors for both OS and PFS, yet not independent. Besides, ITGA3/B4/B6/B7/B8 were highly expressed in OC compared to normal ovarian tissue, ITGA3/A7/B4 were highly expressed in metastasis site compared to primary ovarian cancer, ITGA3/B8 were highly expressed in platinum-based chemotherapy resistance/non-response patients compared to sensitive/response patients. These highlighted the integrins ITGA3/B4/B8 and indicated they may be important in promoting ovarian cancer progression (Supplementary Figure 3B). It was interesting to notice that, the heterodimeric partners ITGA6 and ITGB4 were contrarily predicting prognosis of OS and PFS in OC, the other similar heterodimeric partners were ITGA5 and ITGB1, indicating possible independent functions of single integrin or novel integrin heterodimers under the pathological context of OC, like PPI indicated ITGA3 and ITGB4. Next, to explore the potential function of ITGA3 and ITGB4 in OC, their co-expressed genes (*r* > 0.3) were analyzed by GO (Gene Ontology) and KEGG (Kyoto Encyclopedia of Genes and Genomes) in DAVID (Database for Annotation, Visualization, and Integrated Discovery) database (Figures 6A,B). There were the same GOTERM and KEGG PATHWAY appeared in both ITGA3 and ITGB4 top enrichment lists, such as GO:0005515 (protein binding), GO:0098641 (cadherin binding involved in cell-cell adhesion) and GO:0005200 (structural constituent of cytoskeleton) in molecular function enrichment; GO:0005654 (nucleoplasm) and GO:0005913 (cell-cell adherens junction) in cellular components enrichment; hsa03030 (DNA replication), hsa03040 (Spliceosome), hsa05166 (HTLV-I infection), hsa03430 (Mismatch repair), hsa05222 (Small cell lung cancer), and hsa05200 (Pathways in cancer) in KEGG enrichment. In summary, ITGA3 and ITGB4 might synergistically promote OC through affecting mismatch repair, DNA replication, spliceosome and cell adhesion. It worth to mention ITGA3 associated genes uniquely enriched for hsa04512 (ECM-receptor interaction), hsa04510 (Focal adhesion), hsa04151 (PI3K-Akt signaling pathway) in KEGG (Figure 6A), while ITGB4 associated genes uniquely enriched for has04110 (Cell cycle) in KEGG; GO:0051301 (cell division), GO:0007067 (mitotic nuclear division), GO:0006260 (DNA replication), GO:0007093 (mitotic cell cycle checkpoint) and GO:0006271 (DNA strand elongation involved in DNA replication) in BP; GO:0003678 (DNA helicase activity), GO:0017137 (Rab GTPase binding) and GO:0005096 (GTPase activator activity) in MF (Figure 6B). ITGA3 co-expressed genes mainly enriched for extracellular matrix, but ITGB4 co-expressed genes mainly enriched for cytoplasm and nucleus. These results indicate ITGA3 might be more involved in cell-matrix interaction and trigger extracellular signaling delivery to intracellular; ITGB4 might be important for adhesion and proliferation regulation.

Although ITGA3 and ITGB4 might play important roles in the OC process and chemoresistance, the drug directly targeting ITGA3 or ITGB4 were rare. The only ITGB4 directly targeted drug R1295 was still under investigation for the treatment of rheumatoid arthritis. Basing on text-mining, approved drug tyrosine for antidepressant and investigational drug serine for the natural moisturizing could potentially agonist both ITGA3 and ITGB4. Other drugs like approved drug calcipotriol for the plaque psoriasis, approved drug nitric oxide for the hypoxia respiratory failure, approved drug oxygen for the hypoxemia, approved drug calcium for the nutraceutical and investigational drug for the breakdown of muscle proteins could potentially agonist ITGB4. We also analyzed if they were indirectly druggable through their interaction partner proteins or their regulator microRNA and transcriptional factors by Unihi database (Figure 6C). The nodes in red showed drug targeting interaction partner of ITGA3 and ITGB4, which included GRB2, YES1, VCP, FYN, PRKCA, PRKCD, ITGAL, EGFR, ERBB2, and SMAD2 from ITGB4 partner; ITGB1, HGF, TGM2, ITGB3, CALR, EPHA2, and CD44 from ITGA3 partner; PTK2, ITGB2, MET, and UBC from both of them. The drugs targeted each interaction partners were illuminated in Table 7. It should be noted that the interaction of ITGB2/ITGAL with ITGB4 and ITGB2/ITGB3/CD44 with ITGA3 were generated using a computational text-mining approach. However, protein-protein interaction might be affected by a certain context in the cell, which interaction was accused of the OC process still needed further investigation, and effort of developing drugs directly target ITGA3 and ITGB4 should be taken into account.

**Table 7:** Information for available drugs targeted ITGA3/B4 and interaction partners.

**Gene**	**Interaction partner**	**Drug name**	**DBID**
ITGA3/ITGB4		Tyrosine	DB00135
		Serine	DB00133
ITGB4		R1295	DB05122
		Calcipotriol	DB02300
		Nitric oxide	DB00435
		Oxygen	DB09140
		Calcium	DB01373
		Leucine	DB00149
ITGA3	TGM2	Guanosine-5′-Diphosphate	DB04315
ITGA3	ITGB3	Tirofiban	DB00775
		Abciximab	DB00054
		Eptifibatide	DB00063
		Antithymocyte globulin	DB00098
ITGA3	CALR	Melatonin	DB01065
		Tenecteplase	DB00031
		Antihemophilic factor	DB00025
ITGA3	EPHA2	Dasatinib	DB01254
		Phosphoaminophosphonic Acid-Adenylate Ester	DB04395
ITGA3	CD44	Hyaluronan	DB08818
ITGA3	ITGB1	Antithymocyte globulin	DB00098
ITGA3	HGF	O2-Sulfo-Glucuronic Acid	DB02264
		“N,O6-Disulfo-Glucosamine”	DB03959
ITGA3/ITGB4	MET	K-252a	DB02152
ITGA3/ITGB4	ITGB2	Simvastatin	DB00641
ITGA3/ITGB4	PTK2	2-({5-CHLORO-2-[(2-METHOXY-4-MORPHOLIN-4-YLPHENYL	DB07460
		Adenosine-5′-Diphosphate	DB03431
		7-PYRIDIN-2-YL-N-(3,4,5-TRIMETHOXYPHENYL)-7H-PYRR	DB07248
ITGA3/ITGB4	UBC	N-Formylmethionine	DB04464
ITGB4	SMAD2	Phosphonoserine	DB04522
ITGB4	EGFR	Flavopiridol	DB03496
		Trastuzumab	DB00072
		Erlotinib	DB00530
		Panitumumab	DB01269
		S-{3-[(4-ANILINOQUINAZOLIN-6-YL)AMINO]-3-OXOPROPYL	DB07602
		Lapatinib	DB01259
		Lidocaine	DB00281
		Gefitinib	DB00317
		Cetuximab	DB00002
		N-[4-(3-BROMO-PHENYLAMINO)-QUINAZOLIN-6-YL]-ACRYLA	DB07662
ITGB4	ERBB2	Trastuzumab	DB00072
		Lapatinib	DB01259
ITGB4	VCP	Adenosine-5′-Diphosphate	DB03431
		Phosphoaminophosphonic Acid-Adenylate Ester	DB04395
ITGB4	ITGAL	Lovastatin	DB00227
		Efalizumab	DB00095
		Antithymocyte globulin	DB00098
		LFA703	DB03932
		(S)-2-(S)-3-ISOBUTYL-2,5-DIOXO-4-QUINOLIN-3-YLME	DB04724
		1-Acetyl-4-(4-{4-[(2-Ethoxyphenyl)Thio]-3-Nitrophe	DB02177
ITGB4	PRKCD	13-Acetylphorbol	DB04376
ITGB4	YES1	Dasatinib	DB01254
ITGB4	PRKCA	Phosphatidylserine	DB00144
		Vitamin E	DB00163
ITGB4	FYN	Dasatinib	DB01254
		1-Methoxy-2-[2-(2-Methoxy-Ethoxy]-Ethane	DB02078
ITGB4	GRB2	4-[(10 s,14 s,18 s)-18-(2-Amino-2-Oxoethyl)-14-(1-Naphthylmethyl)−8,17,20-Trioxo-7,16,19-Triazaspiro[5.14]Icos-11-En-10-Yl] Benzylphosphonic Acid	DB03276
		Pegademase	DB00061

### Imbalance of Integrin Heterodimers in Ovarian Cancer

In the previous analysis, we noticed elevated ITGB4 mRNA showed significant association with poor prognosis, metastasis and platinum resistance. However, its heterodimeric partner ITGA6 showed a positive correlation with prognosis and lack significant correlation with metastasis and platinum resistance. Given that the heterodimeric integrin receptor is important for cell-cell and cell-matrix interaction, the disruption of heterodimeric structure in cancer might lead to metastasis. So, we analyzed the correlation of each integrin members in normal ovarian and ovarian cancer.

**Figure 7:**
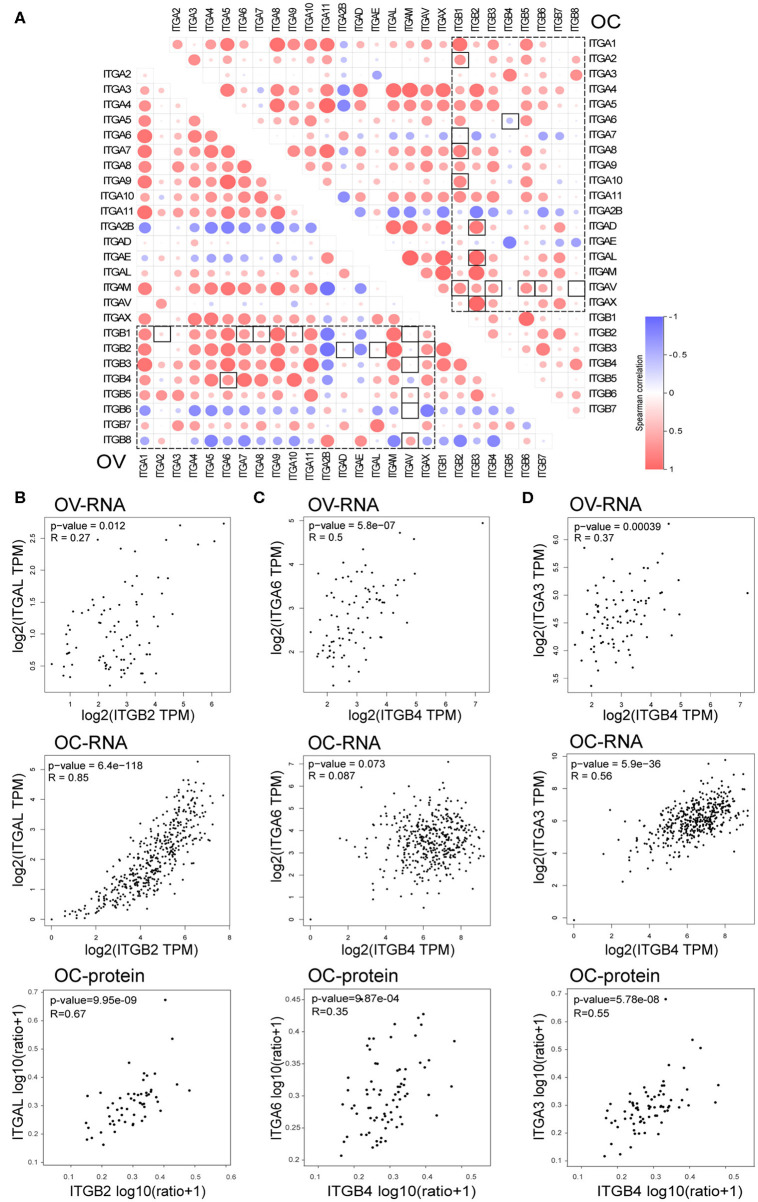
Correlation between integrin genes in OC and normal ovary tissue. **(A)** Correlation between ITGAs and ITGBs mRNA expression in OC and normal ovary tissue (box with dash lines), correlation ratio alterations between OC and normal ovary tissue were highlighted with a solid line frame. **(B)** Correlation of ITGB2 and ITGAL mRNA expression in normal ovary (up) and OC (down) tissue, correlation of ITGB2 and ITGAL protein level in OC tissue (bottom). **(C)** Correlation of ITGB4 and ITGA6 mRNA expression in normal ovary (up) and OC (middle) tissue, correlation of ITGB4 and ITGA6 protein level in OC tissue (bottom). **(D)** Correlation of ITGB4 and ITGA3 mRNA expression in normal ovary (up) and OC (middle) tissue, correlation of ITGB4 and ITGA3 protein level in OC tissue (bottom).

It's well known that integrin formed heterodimeric receptors by interaction between ITGB1 and ITGA1/A2/A3/A4/A5/A6/A7/A8/A9/A10/A11/AV, ITGB2 and ITGAD/AL/AM/AX, ITGB3 and ITGAV/A2B, ITGB7 and ITGA4/AE, ITGB5/B6/B8 and ITGAV, ITGB4 and ITGA6 (Supplementary Figure 3B; Kobayashi et al., 2017). About half of the reported partner mRNA expression correlations obviously changed between normal and malignant ovarian tissue (Figure 7A), and majority of those altered mRNA expression correlation still established in protein level in OC (Supplementary Figure 3C). These changes also occurred in the potential important integrin members identified by our previous analysis. ITGB2 showed high correlations with ITGAD/AL/AM/AX in OC, while ITGB2 showed very low correlations with ITGAD/AL in the normal ovary. The correlation ratio between ITGB2 and ITGAL mRNA expression raised from 0.27 in normal ovary to 0.85 in OC, ITGB2 protein level was also significantly associated ITGAL protein level with a ratio of 0.67 in OC (Figure 7B). In addition, ITGB2 showed tendency toward co-occurrency with ITGAX (Log2 Odds Ratio>3, *p* < 0.001), ITGAM (Log2 Odds Ratio>3, *p* < 0.001), ITGA4 (Log2 Odds Ratio = 2.756, *p* = 0.003) and ITGAL (Log2 Odds Ratio = 2.853, *p* = 0.010) in OC (cBioPortal-TCGA, Firehose Legacy) by mutual exclusivity analysis. This result supported our previous TIL analysis, indicated they were all TILs associated integrins and might function as heterodimeric receptors.

Opposite to TIL associated integrins, we found the mRNA expression correlation ratio between ITGB4 and ITGA6 decreased from 0.5 in the normal ovary to 0.087 in the OC (Figure 7C), which indicated a potential independent function in OC. Accidentally, we found the other integrin alpha subunit ITGA3 which showed elevated mRNA expression in OC and correlated with poor prognosis, metastasis and platinum resistance as well as ITGB4, had a physical protein interaction with ITGB4 as revealed by previous PPI network (Figure 6C). More importantly, the correlation ration between ITGA3 and ITGB4, raised from 0.37 in normal ovary to 0.56 in OC (Figure 7D). Although ITGA6 and ITGA3 showed similar gene alteration percentage (9%), and similar correlation ratio with CNA (0.33 and 0.35 respectively) and protein level (0.74 and 0.69 respectively) in OC (Figure 2B and Supplementary Figure 1). ITGB4 and ITGA3 showed a tendency toward co-occurrency (Log2 Odds Ratio>3, *p* = 0.019), ITGB4 and ITGA6 showed no association (Log2 Odds Ratio = −0.046, *p* = 0.726) in OC (cBioPortal-TCGA, Firehose Legacy) by mutual exclusivity analysis. Besides, the ITGB4 protein level showed a lower correlation ratio (*r* = 0.35) with ITGA6 protein level, and a higher correlation ratio (*r* = 0.55) with ITGA3 protein level (Figures 7C,D). These results together indicated a potential disruption of alpha6beta4 heterodimer and a potential competitively formation of a novel alpha3beta4 heterodimer. Hence, we surmised a critical role of alpha3beta4 heterodimer or distinctly independent functions of ITGB4 and ITGA3 played in ovarian cancer. However, whether the imbalance or reconstruction of heterodimers affects ovarian cancer development of progression still needs further study.

## Discussion

This study demonstrates that the ITGA3 and ITGB4/B6/B7/B8 are highly expressed in OC when compared to normal ovary tissues. Further survival analysis of OC patients indicates increased ITGA6 mRNA positively associated with favored OS and PFS; increased ITGA3/B3/B4/B8 mRNA positively associate with unfavored OS and PFS in OC, especially with advanced OC; higher ITGAE/B7 mRNA associated with better prognosis; while higher ITGAL/AM/B2 mRNA associated with worse prognosis in OC, especially with early OC. ITGA3 and ITGB4 also significantly associated with metastasis and platinum resistance status in OC patients. These may due to their function in regulating cell adhesion and proliferation in OC. Besides, ITGA4/AL/AM/AX and ITGB2/B7 obviously correlate with TILs, particularly strongly correlate with monocyte, M2 macrophage and exhaustion T cells, which usually play pro-tumor and immunosuppressive role in cancer. Our study suggests that integrin members serving different functions in OC, two clusters of integrin may closely connect with OC. One cluster contains ITGA3 and ITGB4, showed direct associations with prognosis, metastasis, and drug resistance, may be independent predictors of advanced OC prognosis and direct molecular therapy targets; another cluster contains ITGAL/AM/B2/B7, correlated across various TILs, may be independent predictors of early OC prognosis and render novel indirect OC therapy targets through modulating the immune response.

Our study identified ITGA3 and ITGB4 as important integrin oncogene in OC. For the first time, we present ITGA3 and ITGB4 were independent predictors associated with poor OS and PFS in OC. Lee et al. and Chen et al. reported ITGB4 mediated mutant p53 (R248; Lee et al., 2015) induced adhesiveness and Shh ligand (Chen et al., 2014) induced migration in OC, which support its role in oncogenic. However, Baldwin et al. reported CD151-alpha3beta1 integrin complex repressed proliferation in OC (Baldwin et al., 2014) and suggested its suppressive role in OC. Mechanically, our analysis showed ITGA3 was mainly enriched for cell-matrix interaction rather than cell cycle or proliferation, it was possible that the impact of ITGA3 on adhesion overwhelmed its impact on suppressing proliferation. ITGB8 was another potential integrin oncogene in OC. Consistent with our results, He et al. found ITGB8 upregulation associated with poor OS and RFS in OC (He et al., 2018); Cui et al. found miR-199a-3p downregulated ITGB8 and resulted in cisplatin sensitivity in OC (Cui et al., 2018).

ITGA6 and ITGA7 were both negatively associated with OS and PFS in OC in the present study. Givant-Horwitz et al. found lower ITGA6 mRNA expression in FIGO stage IV ovarian cancer solid tumors compared to stage III OC, and correlated with shorter OS (Givant-Horwitz et al., 2003). But Villegas-Pineda et al. reported blocking of ITGA6 decreased migration and invasion of SKOV3 cells, as well as partially sensitized SKOV3 cells response to carboplatin (Villegas-Pineda et al., 2017). So far, the study about ITGA7 in OC was blank. Despite the protective prognosis as ITGA7 showed in survival analysis, our study showed ITGA7 mRNA expression increased in metastasis OC tissues. Basing on available data, it's still a far way to conclude their functions in OC.

Our analysis also showed higher ITGA5/A10/A2B and ITGB3 mRNA correlated with unfavored OS in OC. Despite the scarcity of ITGA2B and ITGA10 studies in OC, results from ITGA5 studies in OC were consistently support its role as an oncogene. Li et al. reported increased ITGA5 protein level correlated with advanced OC stage and differentiation degree (Li et al., 2010). Multiple studies reported ITGA5 promotes migration and invasion *in vitro*, as well as metastasis *in vivo* (Sawada et al., 2008; Villegas-Pineda et al., 2017). Its expression and function were regulated by miR-17/miR-92a (Ohyagi-Hara et al., 2013; Gong et al., 2016) and fibronectin-binding (Mitra et al., 2011) respectively. We also observed ITGA5 highly expressed in metastatic OC tissue of GSE30587 (Brodsky et al., 2014). The majority of studies claimed ITGB3 acted as a tumor suppressor, due to serials of survival analysis showed ITGB3 highly expressed in OC survivors (Partheen et al., 2008, 2009; Kaur et al., 2009), siITGB3 in SKOV3 increased cell proliferation, migration, and invasive activity (Chen et al., 2009, 2016; Kaur et al., 2009). While other studies also showed thyroid, KAI or PAX8 could regulate ITGB3 induced proliferation and metastasis (Shinderman-Maman et al., 2016; Soriano et al., 2019), this might be mediated by downstream vitronectin (Ruseva et al., 2009) or ILK (Lössner et al., 2009) signaling. It's possible ITGB3 was a two-edged sword in OC, the cellular context might determine its main function.

In the present study, lower ITGA10/AX and ITGB1 mRNA significantly associated with poor PFS. Unfortunately, there was no study of ITGA10 and ITGAX in OC till now, yet ITGB1 was reported as an oncogene. Davidson et al. claimed the ITGB1 protein level positively correlated with a higher clinical stage (Davidson et al., 2003). Experimental knockdown ITGB1 expression showed impaired migration and invasion via FAK, MMP or MEK signaling (Mitra et al., 2011; Zhang and Zou, 2015), and regulated by AKT, EGF, Oct4A, miR-17 or lncRNA HULC (Arboleda et al., 2003; Lau et al., 2012; Gong et al., 2016; Samardzija et al., 2016; Chen et al., 2017). These seemed inconsistent with our analysis, which showed ITGB1 was lowly expressed in OC from the GSE26712 (Bonome et al., 2008) dataset, and lowly expressed in omental metastasis from the GSE131978 (Tassi et al., 2019) dataset.

Our analysis revealed a group of integrin associated with TILs, including ITGA4/AL/AM/AX and ITGB2/B7. ITGAX was reported to express on DC, mediating DNA delivery and DC maturation via cytosolic cGAS/STIN DNA-sensing pathway (Fyrstenberg Laursen et al., 2019). ITGAM highly expressed on myeloid cell subsets and plays an important role in trafficking and cellular functions in inflamed tissues (Panni et al., 2019). ITGAL expressed on NK cell (Haspels et al., 2018) and T cell, mediate T cell migration by responding to direct CLL cell-contact induced Rho GTPase signaling suppression (Ramsay et al., 2013) and FAK inhibited ITGA4 activation (Cantor et al., 2015). And Gardner et al. reported ITGA4 expression was not detected in OC cell lines (Gardner et al., 1995). But our results showed decreased ITGA4 mRNA significantly associate with poor PFS, this might owe to its function in the immune response.

ITGB2 could form heterodimeric receptors with ITGAD/AL/AM/AX, so it was also expressed on DC, myeloid cell subsets, NK cells and T cells (Dadaglio et al., 2003; Cantor et al., 2015; Haspels et al., 2018; Panni et al., 2019). A bioinformatics analysis showed ITGAM and ITGB2 might be critical for OC metastasis (O'Shannessy et al., 2015). ITGB2/FAK pathway could promote OC growth and proliferation *in vitro* and *in vivo*, and could be targeted by bufalin (Li et al., 2018). These studies indicated ITGB2 might present in multiple cell types in the heterogeneous tumor, and increased the complexity of ITGB2. We found ITGB2 highly expressed in OC from the GEPIA database and highly expressed in metastatic OC in GSE30587 (Brodsky et al., 2014) datasets, which support its role in promoting OC progression. ITGB7 also reported to express in both immune cells and tumor cells, but its study in OC was vacancy. ITGB7 expression in immune cells were usually paired with ITGAE (CD103), especially in the residing memory CD8+ T cells (Le Floc'h et al., 2007; Anz et al., 2011; Franciszkiewicz et al., 2013). ITGAE highly expression accompany with high infiltrating T cells, was strongly associated with favored OS in HGSC (Webb et al., 2014a). Furthermore, CD103(+) CD8 TIL express PD-1 and appear quiescent in the HGSC tumor microenvironment, retain functional competence and demonstrate strong prognostic significance (Webb et al., 2014b). These were consistent with our analysis which showed high ITGAE and ITGB7 mRNA associate with favored OS in OC.

Our study has some limitations. First, the present study relies on the public databases, which are constantly supervised and extended, it's possible to affect the outcome of the study. Second, the analysis of the integrin family in OC is limited in the mRNA expression level, while proteins are the actual function executor. Third, this study lacks the *in vitro* and *in vivo* experiment to confirm the function of integrins in OC. Hence, further studies are necessary to validate current findings and conjectures.

In summary, our results highlight the neglected integrin members ITGA3, ITGB4 and ITGB8, and speculate that they are important oncogene and independent prognostic factors in OC. This study also calls attention for ITGAL/AM/B2/B7 in immune response of OC, might offer clues to improve immunotherapy of OC.

## Data Availability Statement

The datasets presented in this study can be found in online repositories. The names of the repository/repositories and accession number(s) can be found in the article/Supplementary Material.

## Ethics Statement

Our study protocol was approved by the Ethics Committee of the Xiangya Medical College of Central South University. All data were retrieved from the online database, it was confirmed that all written informed consent had been obtained.

## Author Contributions

AW and SZ designed the research. AW, SZ, JL, YH, and WD retrieved and analyzed the data, AW and SZ wrote the paper. GS and GY supervised the analysis and revised the paper. All authors contributed to the article and approved the submitted version.

## Conflict of Interest

The authors declare that the research was conducted in the absence of any commercial or financial relationships that could be construed as a potential conflict of interest.
