# Supplementary material for: Integrated Analysis of Prognostic and Immune Associated Integrin Family in Ovarian Cancer
Source: Front Genet. 2020 Jul 17;11:705. doi: 10.3389/fgene.2020.00705 (PMC7379341; doi:10.3389/fgene.2020.00705)
Supplement: Supplementary file 1 [file Table_1.docx]

Supplementary table 1. Prognostic integrin genes validation in chemotherapy-received patients in TCGA and external GEO dataset.

| Probe ID | Gene Symbol |  | TCGA (all), N=557 | | |  | TCGA (chemotherapy), N=505 | | |  | External dataset validation | | | |  |
| --- | --- | --- | --- | --- | --- | --- | --- | --- | --- | --- | --- | --- | --- | --- | --- |
|  |  |  | HR | 95% CI | *p*-value |  | HR | 95% CI | *p*-value |  | Accession ID | HR | 95% CI | *p*-value | |
| A. OS |  |  |  |  |  |  |  |  |  |  |  |  |  |  | |
| 201474_s_at | ITGA3 |  | 1.3 | 1.03-1.64 | **0.027** |  | 1.26 | 0.98-1.61 | 0.068 |  | GSE30161, N=58 | 2.9 | 1.11-7.57 | **0.023** | |
| 201389_at | ITGA5 |  | 1.29 | 1.02-1.64 | **0.033** |  | 1.3 | 1.01-1.68 | **0.043** |  |  |  |  |  | |
| 201656_at | ITGA6 |  | 0.73 | 0.58-0.93 | **0.009** |  | 0.72 | 0.56-0.93 | **0.01** |  | GSE29163, N=107 | 0.58 | 0.25-0.94 | **0.027** | |
| 209663_s_at | ITGA7 |  | 0.78 | 0.62-0.98 | **0.036** |  | 0.8 | 0.62-1.02 | 0.071 |  |  |  |  |  | |
| 206766_at | ITGA10 |  | 1.29 | 1.02-1.63 | **0.031** |  | 1.23 | 0.96-1.59 | 0.10 |  | GSE14764, N=80 | 3.44 | 1.37-8.64 | **0.0052** | |
| 216956_s_at | ITGA2B |  | 1.42 | 1.09-1.85 | **0.0081** |  | 1.31 | 0.99-1.73 | 0.054 |  | GSE29163, N=107 | 1.64 | 1.04-2.58 | **0.03** | |
| 205055_at | ITGAE |  | 0.76 | 0.61-0.97 | **0.024** |  | 0.69 | 0.54-0.89 | **4.20E-03** |  |  |  |  |  | |
| 204626_s_at | ITGB3 |  | 1.41 | 1.12-179 | **0.0037** |  | 1.46 | 1.13-1.9 | **3.60E-03** |  |  |  |  |  | |
| 214292_at | ITGB4 |  | 1.38 | 1.07-1.78 | **0.013** |  | 1.37 | 1.04-1.81 | **0.023** |  | GSE30161, N=58 | 2.1 | 1.06-4.15 | **0.03** | |
| 205718_at | ITGB7 |  | 0.75 | 0.56-1 | **0.047** |  | 0.77 | 0.56-1.05 | 0.09 |  |  |  |  |  | |
| 211488_s_at | ITGB8 |  | 1.39 | 1.08-1.78 | **0.01** |  | 1.57 | 1.19-2.06 | **1.10E-03** |  | GSE30161, N=58 | 2.55 | 1.3-5 | **0.0046** | |
| B. PFS |  |  |  |  |  |  |  |  |  |  |  |  |  |  | |
| 201474_s_at | ITGA3 |  | 1.27 | 1-1.6 | **0.049** |  | 1.28 | 1.01-1.62 | **0.043** |  | GSE30161, N=54 | 2.27 | 1.15-4.49 | **0.016** | |
|  |  |  |  |  |  |  |  |  |  |  | GSE14764, N=80 | 1.81 | 1.06-3.11 | **0.028** | |
| 213416_at | ITGA4 |  | 0.68 | 0.53-0.87 | **0.0019** |  | 0.68 | 0.53-0.87 | **1.90E-03** |  |  |  |  |  | |
| 201656_at | ITGA6 |  | 0.63 | 0.5-0.79 | **6.50E-05** |  | 0.62 | 0.49-0.79 | **5.40E-05** |  | GSE29163, N=107 | 0.55 | 0.34-0.89 | **0.014** | |
| 209663_s_at | ITGA7 |  | 0.7 | 0.56-0.88 | **0.0021** |  | 0.7 | 0.56-0.89 | **2.60E-03** |  | GSE30161, N=54 | 0.5 | 0.26-0.97 | **0.036** | |
| 206766_at | ITGA10 |  | 0.75 | 0.59-0.95 | **0.016** |  | 0.76 | 0.6-0.97 | **0.024** |  |  |  |  |  | |
| 210184_at | ITGAX |  | 0.77 | 0.6-0.99 | **0.043** |  | 0.8 | 0.63-1 | 0.05 |  |  |  |  |  | |
| 215878_at | ITGB1 |  | 0.76 | 0.6-0.95 | **0.018** |  | 0.77 | 0.61-0.98 | **0.03** |  |  |  |  |  | |
| 214292_at | ITGB4 |  | 1.37 | 1.08-1.74 | **0.01** |  | 1.37 | 1.08-1.74 | **0.0087** |  |  |  |  |  | |
| 208083_s_at | ITGB6 |  | 1.28 | 0.98-1.67 | 0.072 |  | 1.32 | 1.01-1.74 | **0.044** |  | GSE30161, N=54 | 1.85 | 1-3.39 | **0.045** | |
| 211488_s_at | ITGB8 |  | 1.48 | 1.15-1.91 | **0.0023** |  | 1.58 | 1.22-2.05 | **4.90E-04** |  | GSE30161, N=54 | 2.66 | 1.42-4.97 | **0.0015** | |
|  |  |  |  |  |  |  |  |  |  |  | GSE14764, N=80 | 1.99 | 1.18-3.35 | **0.0083** | |

Bold font indicates significant difference
