# Supplementary material for: Integrated Analysis of Prognostic and Immune Associated Integrin Family in Ovarian Cancer
Source: Front Genet. 2020 Jul 17;11:705. doi: 10.3389/fgene.2020.00705 (PMC7379341; doi:10.3389/fgene.2020.00705)
Supplement: Supplementary file 2 [file Table_2.docx]

Supplementary table 2. Particular integrin genes mRNA expression association with clinical parameters

| Clinical parameters N(%) |  | ITGA3 | | | | |  | ITGA6 | | |  | ITGB4 | | |  | ITGB8 | | |
| --- | --- | --- | --- | --- | --- | --- | --- | --- | --- | --- | --- | --- | --- | --- | --- | --- | --- | --- |
|  |  | Low | | High | | *p*-value |  | Low | High | *p*-value |  | Low | High | *p*-value |  | Low | High | *p*-value |
| Age |  |  | |  | | 0.869 |  |  |  | 0.837 |  |  |  | 0.195 |  |  |  | 0.548 |
| >=60 |  | 54(47%) | | 124(48%) | |  |  | 37(52%) | 162(53%) |  |  | 55(47%) | 141(55%) |  |  | 141(53%) | 55(50%) |  |
| <60 |  | 61(53%) | | 135(52%) | |  |  | 34(48%) | 141(47%) |  |  | 61(53%) | 117(45%) |  |  | 123(47%) | 55(50%) |  |
| Race |  |  | |  | | 0.139 |  |  |  | 0.493 |  |  |  | 0.266 |  |  |  | 0.527 |
| White |  | 104(93%) | | 220(88%) | |  |  | 60(87%) | 264(90%) |  |  | 103(92%) | 221(88%) |  |  | 225(89%) | 99(91%) |  |
| Others |  | 8(7) | | 31(12%) | |  |  | 9(13%) | 30(10%) |  |  | 9(8%) | 30(12%) |  |  | 29(11%) | 10(9%) |  |
| Ethnicity |  |  | |  | | 0.313 |  |  |  | 0.343 |  |  |  | 0.641 |  |  |  | 0.609 |
| Hispanic or Latino |  | 4(5%) | | 4(3%) | |  |  | 3(6%) | 5(3%) |  |  | 2(3%) | 6(4%) |  |  | 5(3%) | 3(5%) |  |
| Others |  | 70(95%) | | 143(97%) | |  |  | 49(94%) | 164(97%) |  |  | 70(97%) | 143(96%) |  |  | 151(97%) | 62(95%) |  |
| Stage |  |  | |  | | 0.180 |  |  |  | 0.932 |  |  |  | 0.373 |  |  |  | 0.082 |
| Ⅰ+Ⅱ |  | 4(3%) | | 18(7%) | |  |  | 4(6%) | 18(6%) |  |  | 5(4%) | 17(7%) |  |  | 12(5%) | 10(9) |  |
| Ⅲ+Ⅳ |  | 111(97%) | | 238(93) | |  |  | 66(94%) | 283(94%) |  |  | 111(96%) | 238(93%) |  |  | 251(95%) | 98(91%) |  |
| Grade |  |  | |  | | 0.240 |  |  |  | 0.950 |  |  |  | 0.885 |  |  |  | 0.918 |
| G1+G2 |  | 10(9%) | | 33(13%) | |  |  | 8(12%) | 35(12%) |  |  | 14(12%) | 29(12%) |  |  | 31(12%) | 12(12%) |  |
| G3+G4 |  | 103(91%) | | 218(87%) | |  |  | 61(88%) | 260(88%) |  |  | 101(88%) | 220(88%) |  |  | 229(88%) | 92(88%) |  |
| OS_Status |  |  | |  | | 0.111 |  |  |  | **0.026** |  |  |  | 0.068 |  |  |  | 0.612 |
| Alive |  | 51(44%) | | 92(36%) | |  |  | 19(27%) | 124(41%) |  |  | 52(45%) | 91(35%) |  |  | 103(39%) | 40(36%) |  |
| Dead |  | 64(56%) | | 166(64%) | |  |  | 52(73%) | 178(59%) |  |  | 63(55%) | 167(65%) |  |  | 160(61%) | 70(64%) |  |
| PFS_Status |  |  | |  | | 0.129 |  |  |  | 0.298 |  |  |  | 0.549 |  |  |  | 0.531 |
| Disease_free |  | 32(32%) | | 60(29%) | |  |  | 14(25%) | 78(31%) |  |  | 31(32%) | 61(29%) |  |  | 64(28%) | 28(33%) |  |
| Recurrence/Progression |  | 67(68%) | | 150(71%) | |  |  | 41(75%) | 176(69%) |  |  | 67(68%) | 150(71%) |  |  | 161(72%) | 56(67%) |  |
| Cancer_Status |  |  | |  | | 0.285 |  |  |  | 0.339 |  |  |  | **0.042** |  |  |  | 0.648 |
| Tumor_Free |  | 31(30%) | | 54(24%) | |  |  | 13(21%) | 72(27%) |  |  | 34(33%) | 51(22%) |  |  | 63(26%) | 22(24%) |  |
| With_Tumor |  | 74(70%) | | 171(76%) | |  |  | 49(79%) | 196(73%) |  |  | 69(67%) | 176(78%) |  |  | 176(74%) | 70(76%) |  |
| Chemotherapy |  |  | |  | | 0.218 |  |  |  | 0.813 |  |  |  | 0.399 |  |  |  | **0.020** |
| NO |  | 6(5%) | | 23(9%) | |  |  | 6(8%) | 23(8%) |  |  | 7(6%) | 22(9%) |  |  | 15(6%) | 14(13%) |  |
| YES |  | 109(95%) | | 235(91%) | |  |  | 65(92%) | 279(92%) |  |  | 109(94%) | 235(91%) |  |  | 249(94%) | 96(87%) |  |
| HormoneTherapy |  |  | |  | | 0.840 |  |  |  | 0.247 |  |  |  | 0.541 |  |  |  | 0.120 |
| NO |  | 104(90%) | | 235(91%) | |  |  | 62(87%) | 277(92%) |  |  | 107(92%) | 232(90%) |  |  | 236(89%) | 103(94%) |  |
| YES |  | 11(10%） | | 23(9%) | |  |  | 9(13%) | 25(8%) |  |  | 9(8%) | 25(10%) |  |  | 28(11%) | 6(6%) |  |
| ImmunoTherapy |  |  | |  | | 0.286 |  |  |  | 0.480 |  |  |  | 0.702 |  |  |  | 0.414 |
| NO |  | 110(96%) | | 252(98%) | |  |  | 68(96%) | 294(97%) |  |  | 112(97%) | 250(97%) |  |  | 255(97%) | 107(98%) |  |
| YES |  | 5(4%) | | 6(2%) | |  |  | 3(4%) | 8(3%) |  |  | 4(3%) | 7(3%) |  |  | 9(3%) | 2(2%) |  |
| TargetedTherapy |  |  | |  | | 0.643 |  |  |  | 0.289 |  |  |  | 0.918 |  |  |  | 0.289 |
| NO |  | 106(92%) | | 234(91%) | |  |  | 67(94%) | 273(90%) |  |  | 106(91%) | 234(91%) |  |  | 238(90%) | 102(94%) |  |
| YES |  | 9(8%) | | 24(9%) | |  |  | 4(6%) | 29(10%) |  |  | 10(9%) | 23(9%) |  |  | 26(10%) | 7(6%) |  |
| Primary_therapy_outcome | | |  | | **<0.0001** | |  |  |  | 0.247 |  |  |  | 0.686 |  |  |  | 0.563 |
| Response |  | 82(49%) | | 170(84%) | |  |  | 44(79%) | 208(85%) |  |  | 80(82%) | 172(84%) |  |  | 185(84%) | 67(82%) |  |
| Nonresponse |  | 85(51%) | | 33(16%) | |  |  | 12(21%) | 37(15%) |  |  | 17(18%) | 32(16%) |  |  | 34(16%) | 15(18%) |  |
| Tumor_Residual |  |  | |  | | 0.515 |  |  |  | 0.519 |  |  |  | **0.024** |  |  |  | 0.162 |
| No Macroscopic |  | 17(17%) | | 47(20%) | |  |  | 14(22%) | 50(19%) |  |  | 27(27%) | 37(16%) |  |  | 50(21%) | 14(15%) |  |
| Macroscopic |  | 82(83%) | | 185(80%) | |  |  | 49(78%) | 218(81%) |  |  | 74(73%) | 193(84%) |  |  | 185(79%) | 82(85%) |  |

Bold font indicates significant difference
