# Supplementary figures and images for: Integrated Analysis of Prognostic and Immune Associated Integrin Family in Ovarian Cancer
Source: Front Genet. 2020 Jul 17;11:705. doi: 10.3389/fgene.2020.00705 (PMC7379341; doi:10.3389/fgene.2020.00705)

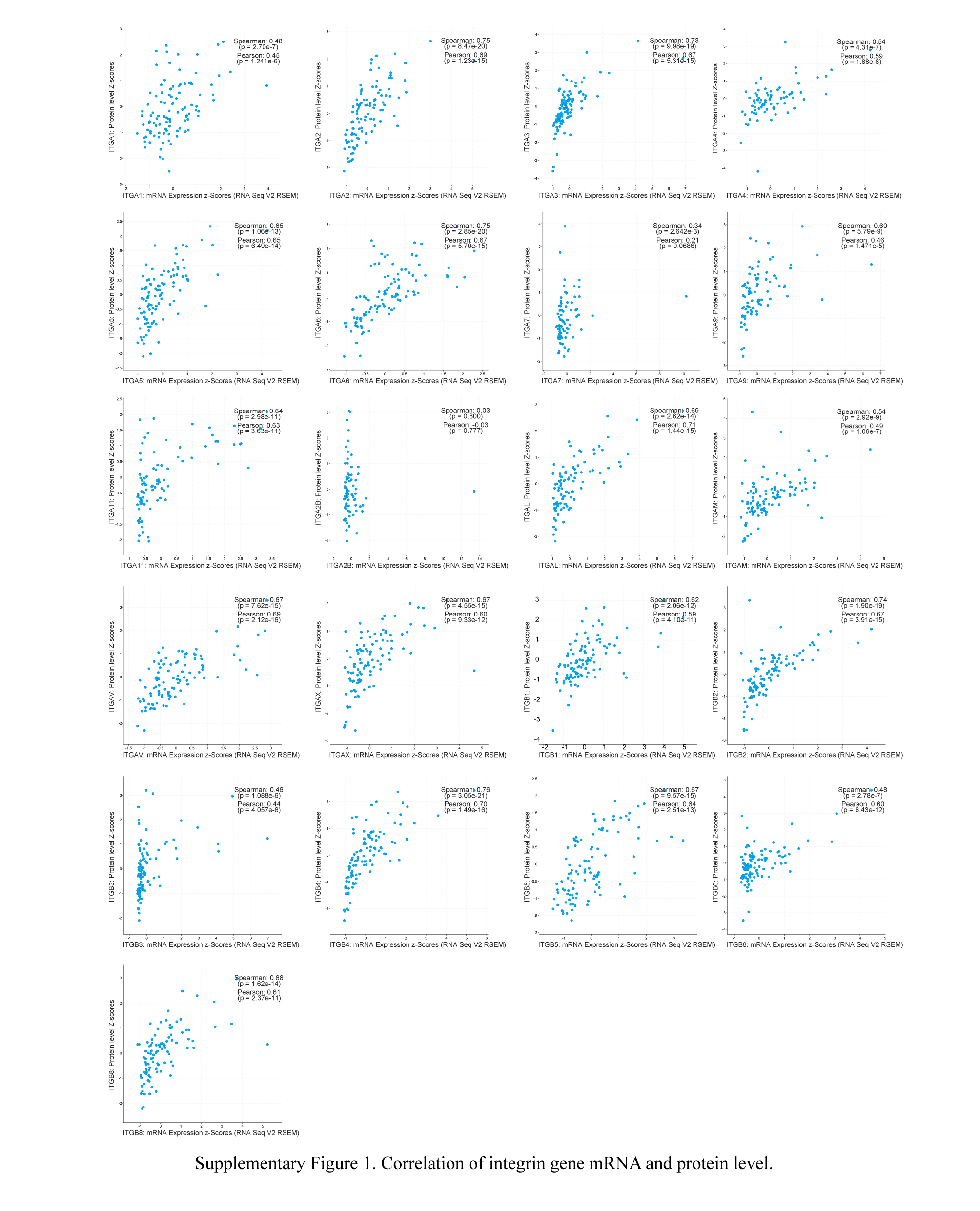

Supplement: Supplementary file 3 [file Image_1.TIF]

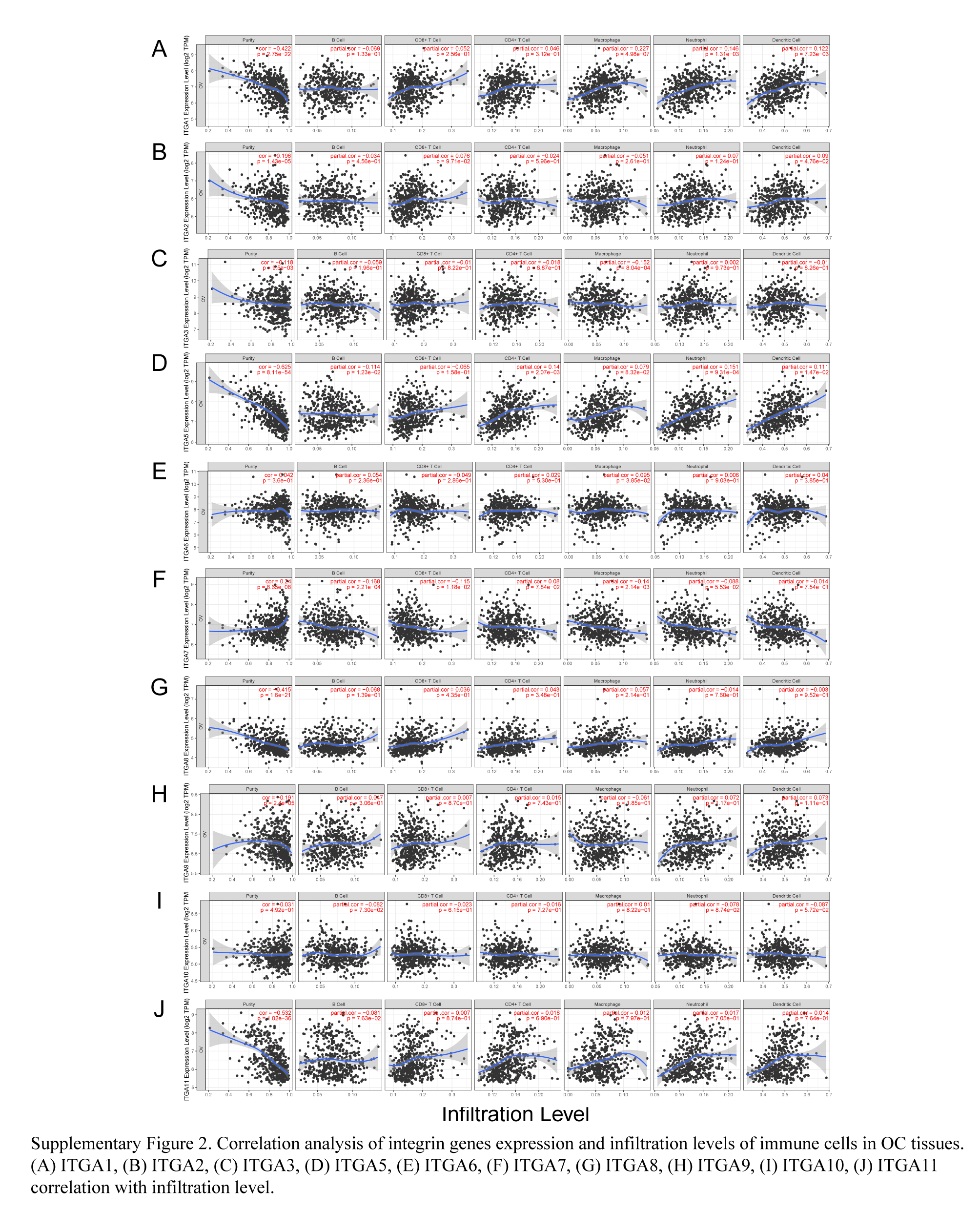

Supplement: Supplementary file 4 [file Image_2.TIF]

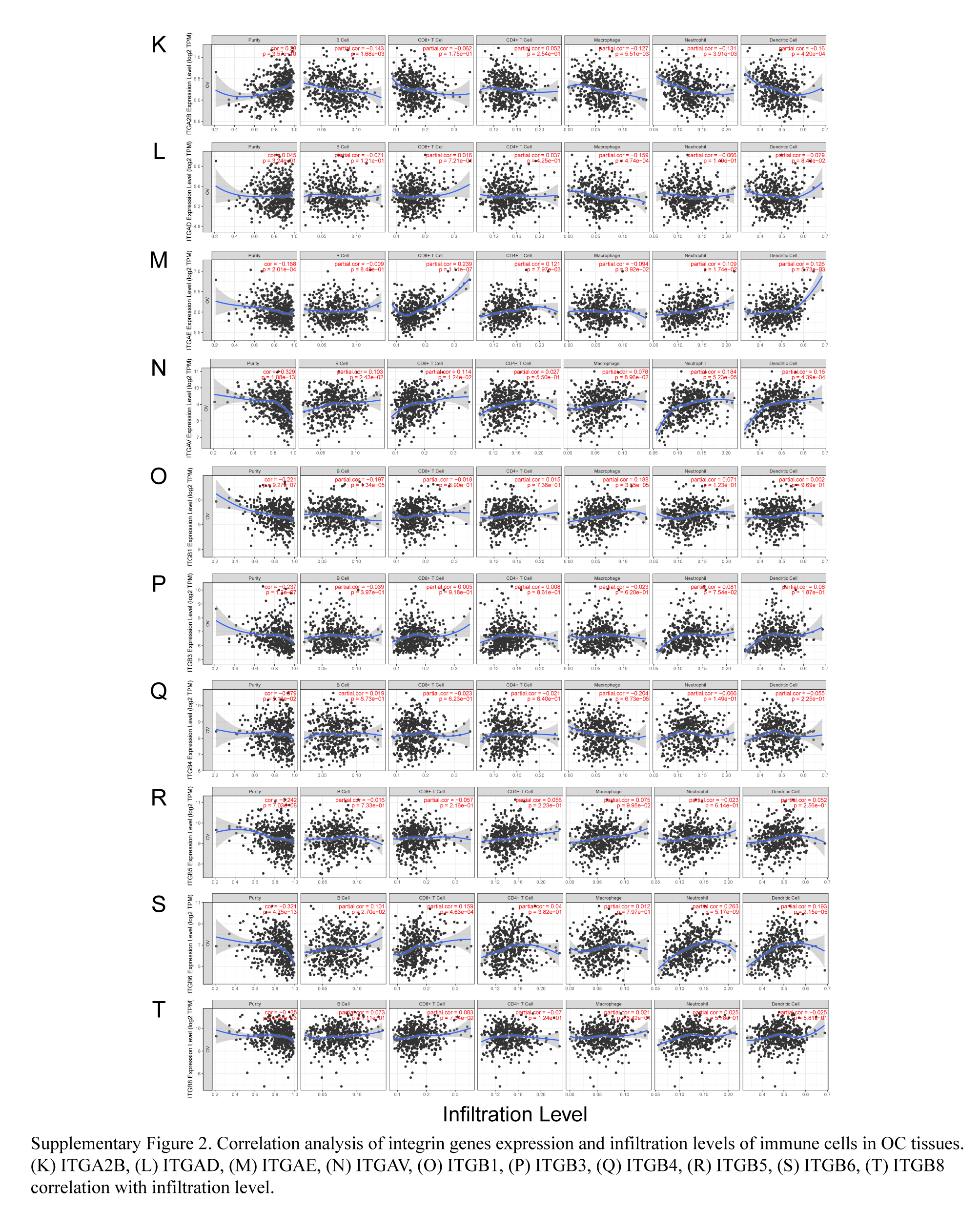

Supplement: Supplementary file 5 [file Image_3.TIF]

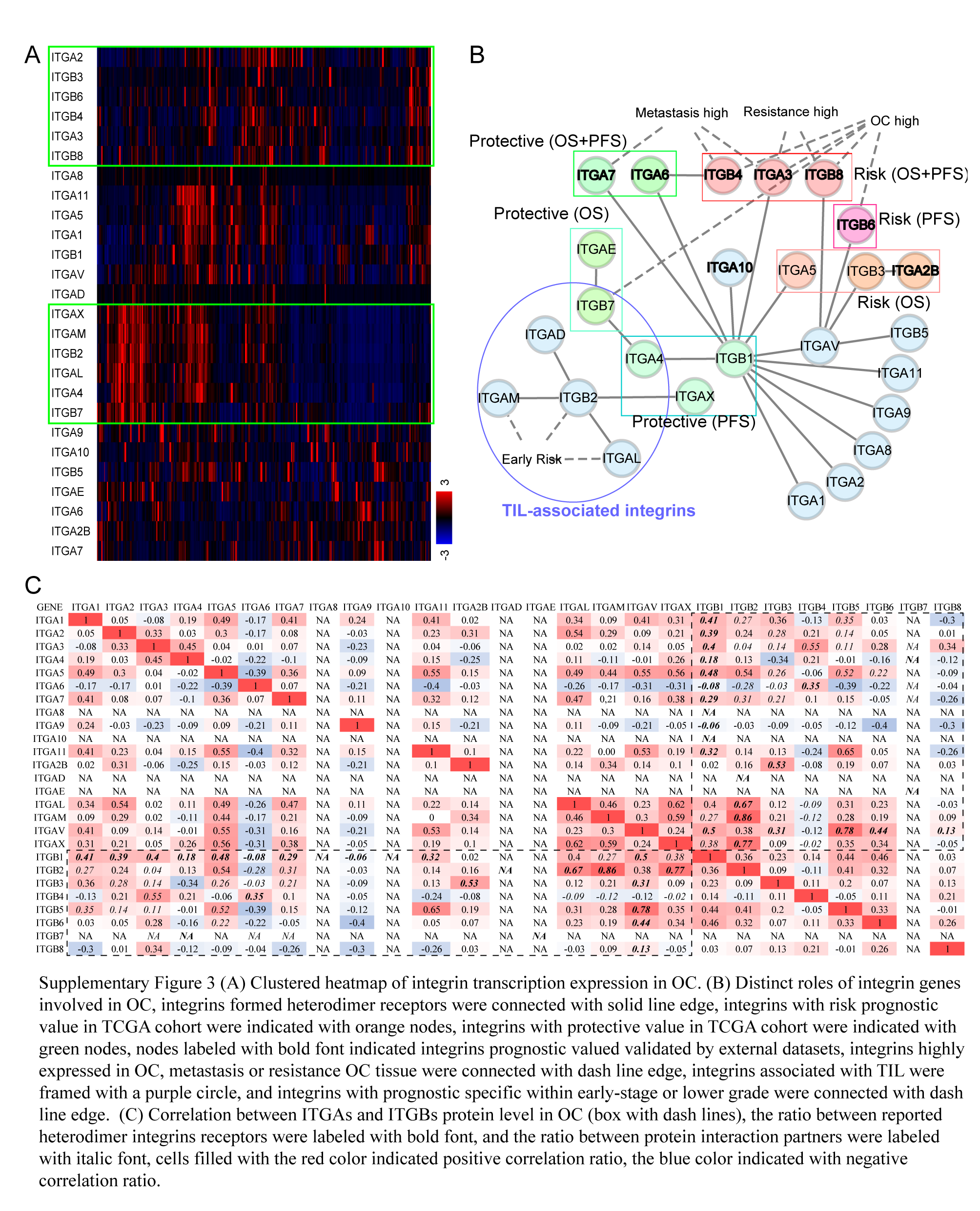

Supplement: Supplementary file 6 [file Image_4.TIF]
